# Targeted Suppression of Lipoprotein Receptor LSR in Astrocytes Leads to Olfactory and Memory Deficits in Mice

**DOI:** 10.3390/ijms23042049

**Published:** 2022-02-12

**Authors:** Aseel El Hajj, Ameziane Herzine, Gaetano Calcagno, Frédéric Désor, Fathia Djelti, Vincent Bombail, Isabelle Denis, Thierry Oster, Catherine Malaplate, Maxime Vigier, Sandra Kaminski, Lynn Pauron, Catherine Corbier, Frances T. Yen, Marie-Claire Lanhers, Thomas Claudepierre

**Affiliations:** 1 UR AFPA Laboratory, Qualivie Team, University of Lorraine, 54505 Vandoeuvre-lès-Nancy, France; ameziane.herzine@univ-lorraine.fr (A.H.); frederic.desor@univ-lorraine.fr (F.D.); fathia.djelti@univ-lorraine.fr (F.D.); thierry.oster@univ-lorraine.fr (T.O.); catherine.malaplate-armand@univ-lorraine.fr (C.M.); maxime.vigier@univ-lorraine.fr (M.V.); lynn.pauron@univ-lorraine.fr (L.P.); catherine.corbier@univ-lorraine.fr (C.C.); frances.yen-potin@inserm.fr (F.T.Y.); marie-claire.lanhers@univ-lorraine.fr (M.-C.L.); 2 UR 7300, Stress Immunity Pathogens Laboratory, Faculty of Medicine, University of Lorraine, 54500 Vandœuvre-lès-Nancy, France; gaetano.calcagno@univ-lorraine.fr (G.C.); sandra.kaminski@univ-lorraine.fr (S.K.); 3 UMR 914, Physiology of Nutrition and Feeding Behaviour, INRAE-Agroparistech-Université Paris-Saclay, 78352 Jouy-en-Josas, France; vincent.bombail@inrae.fr (V.B.); isabelle.denis@inrae.fr (I.D.)

**Keywords:** glia, cholesterol, olfaction, memory, astroglial activation, Alzheimer’s disease

## Abstract

Perturbations of cholesterol metabolism have been linked to neurodegenerative diseases. Glia–neuron crosstalk is essential to achieve a tight regulation of brain cholesterol trafficking. Adequate cholesterol supply from glia via apolipoprotein E-containing lipoproteins ensures neuronal development and function. The lipolysis-stimulated lipoprotein receptor (LSR), plays an important role in brain cholesterol homeostasis. Aged heterozygote *Lsr+/−* mice show altered brain cholesterol distribution and increased susceptibility to amyloid stress. Since LSR expression is higher in astroglia as compared to neurons, we sought to determine if astroglial LSR deficiency could lead to cognitive defects similar to those of Alzheimer’s disease (AD). Cre recombinase was activated in adult *Glast-CreERT/lsrfl/fl* mice by tamoxifen to induce astroglial *Lsr* deletion. Behavioral phenotyping of young and old astroglial *Lsr* KO animals revealed hyperactivity during the nocturnal period, deficits in olfactory function affecting social memory and causing possible apathy, as well as visual memory and short-term working memory problems, and deficits similar to those reported in neurodegenerative diseases, such as AD. Furthermore, GFAP staining revealed astroglial activation in the olfactory bulb. Therefore, astroglial LSR is important for working, spatial, and social memory related to sensory input, and represents a novel pathway for the study of brain aging and neurodegeneration.

## 1. Introduction

Cholesterol transport and homeostasis are tightly regulated in the central nervous system (CNS) to ensure proper development and functioning of the brain throughout its lifetime. However, during normal aging, cholesterol homeostasis is modified. Brain cholesterol levels change in a region-specific manner, especially in the hippocampus [1] and the cortex [2], leading to a moderate reduction in total cholesterol in the brain. These changes could influence the cortical lipid raft composition during aging [3], thus affecting the synaptic functions and normal aging process of the brain [4]. Indeed, many studies have demonstrated the links between cholesterol perturbation and neurodegenerative diseases, including Alzheimer’s disease (AD) [5,6]. Due to the presence of the blood–brain barrier (BBB) [7], the brain must rely on de novo synthesis for cholesterol [8]. Astrocytes provide neurons with cholesterol in the form of lipoproteins, which are primarily apolipoprotein (Apo)E and ApoJ containing high-density lipoprotein-like (HDL-like) particles [9]. These HDL-like particles are exported from the astrocytes via ATP-binding cassette A1 (ABCA1) and ATP-binding cassette subfamily G member 1 (ABCG1) transporters [10], where they then bind to lipoprotein receptors via ApoE and are internalized into target cells through receptor-mediated transport processes.

Lipoproteins and their receptors are key elements ensuring the tight control of cholesterol trafficking. One of the lipoprotein receptors found in the CNS is the lipolysis-stimulated lipoprotein receptor (LSR). The LSR is a multimeric protein complex composed of three subunits (α, α’, β), which are activated in the presence of free fatty acids, thereby revealing a binding site that recognizes ApoB and ApoE [11]. It was first discovered in the liver [12], and later its expression in the CNS was shown [13]. The LSR plays a role in the clearance of ApoB- and ApoE-containing lipoproteins, and helps to maintain normal levels of cholesterol and triglycerides, thereby regulating tissue lipid distribution [12,14]. The consequences of complete *Lsr* inactivation cannot be studied as it provokes lethality at the embryonic stage, most likely due to brain-localized hemorrhages and a leaky BBB [15,16]. In vivo studies on aged *Lsr+/−* mice suggest that reduced LSR levels are associated with cognitive disturbances related to reactivity to novel environments [13]. A significant decrease in lipid droplets, which are lipid-rich cellular organelles that regulate the storage and hydrolysis of neutral lipids, including cholesterol [17], was observed in Purkinje cells of the cerebellum, together with an accumulation of filipin-labeled cholesterol in the neuronal membranes of the hippocampus, in aged *Lsr +/−* mice [13]. We recently detailed the regional expression profile of LSR subunits within the CNS and found specific age-induced reductions in LSR protein expression mainly in the hypothalamus, hippocampus, and olfactory bulb [18]. Moreover, we established that astroglial cells do express LSR at levels higher than those found in neuronal cells [18].

To decipher the role of astroglial LSR in the cholesterol crosstalk within the CNS, we used a conditional Cre/lox recombination system developed in a astroglia-specific transgenic mouse line that allows for temporally controlled site-specific recombination [19]. This approach depends on cell-specific expression of tamoxifen (TAM)-dependent CreERT2 recombinase. The Glast-CreERT2 (Cre) mouse line shows highest Cre-mediated recombination in astroglial cells from the cerebellum, hippocampus, and olfactory bulb, with lower activity in other brain regions and the retina [19]. In the present study, we used the mouse strain Tg(Slc1a3-cre/ERT2)45-72Fwp to suppress *Lsr* in astroglial cells at the age of 2 months following tamoxifen (TAM) treatment, to create the TAM-induced Glast-Cre *lsrfl/fl* (cKO) mouse model. Behavioral phenotyping to assess activity, olfaction, vision, sociability, and short- and long-term memory revealed a series of traits resembling AD. Our findings therefore demonstrate that astroglial *Lsr* disruption is sufficient to switch from normal to pathological aging of the CNS.

## 2. Results

### 2.1. Lsr Deletion and Downregulation of Cholesterol-Related Gene Expression in cKO Mice

**Figure 1:**
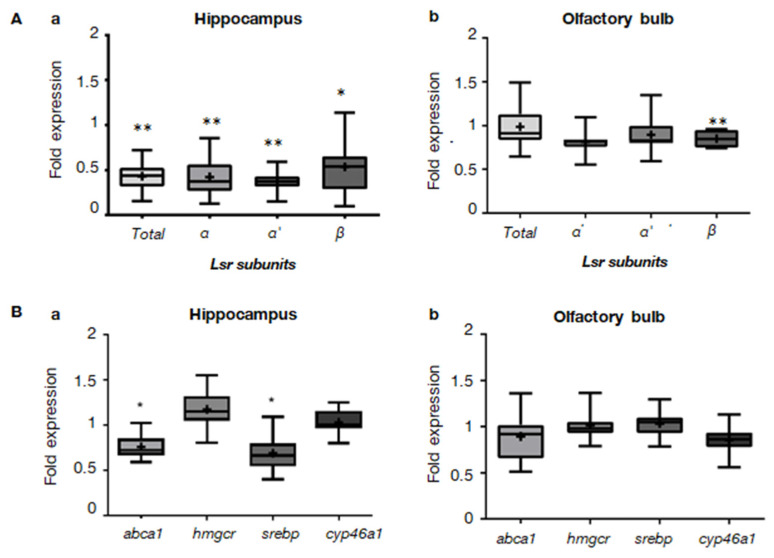
Modification of gene expression in astroglial Lsr cKO mice. (**A**) Verification of *Lsr* gene excision in astroglial cells using RT-qPCR. Box plot presentation of fold expression of total *Lsr* and different *Lsr* subunits α, α’, and β in 13-month-old cKO mice (*n* = 5) with respect to WT mice (*n* = 5) in the hippocampus (**a**) and olfactory bulb (**b**). (**B**) Box plot presentation of fold expression of cholesterol-related gene expression in 13-month-old cKO mice (*n* = 5) with respect to WT mice (*n* = 5) in the hippocampus (**a**) and olfactory bulb (**b**). Abbreviations: *abca1*, ATP-binding cassette transporter 1; *hmgcr*, HMG CoA reductase; *srebp1*, sterol regulatory element-binding transcription factor 1; *cyp46a1*, cytochrome P450 family 46 subfamily A member 1. Statistical significance between WT and cKO mice is shown as: * *p* ≤ 0.05, ** *p* ≤ 0.01.

The Cre mice were crossed with *lsrfl*/*fl* mice to produce Cre-*lsrfl*/*fl (*Supplementary Figure S1). Two-month-old mice were treated with tamoxifen for 5 days, followed by a battery of behavioral tests at different ages (Supplementary Figure S2). No significant differences in body mass were observed between the wild-type controls (WT) and cKO groups, nor was any mortality observed during the experimental period (Supplementary Figure S3). In order to confirm the excision of the floxed *Lsr* gene and the consequent cholesterol-related gene expression, we focused on brain structures exhibiting strong CRE recombinase expression in the transgenic mouse line [19], together with strong LSR expression [18]. Expression levels of *Lsr* mRNA were measured by RT-qPCR on total RNA fractions extracted from different brain region homogenates of 3- and 13-month-old male WT and cKO mice. In the hippocampus, total *Lsr* mRNA was downregulated to 0.627 ± 0.048-fold (*p* = 0.0014) when compared to WT mice at 3 months of age (Supplementary Figure S4), and to 0.385 ± 0.053-fold (*p* = 0.004) at 13 months of age (Figure 1Aa). This was also the case for *Lsr* α (0.354 ± 0.059-fold, *p* = 0.004), *Lsr* α’ (0.337 ± 0.039-fold, *p* = 0.004), and *Lsr* β (0.425 ± 0.083-fold, *p* = 0.035) subunits. This indicates that the *Lsr* gene was successfully excised from the GLAST-expressing astroglial cells, which was retained 11 months after TAM injections. In the olfactory bulb, a significant reduction was observed at 3 months for total *Lsr* mRNA (0.715 ± 0,014-fold, *p* = 0.0004, Supplementary Figure S4). At 13 months of age, a significant reduction was only observed for the *Lsr* β subunit (0.85 ± 0.32-fold, *p* = 0.008), whereas the expression of the other *Lsr* subunits and total RNA remained unchanged (Figure 1Ab). In the cerebellum, *Lsr* RNA (total and subunits) remained unchanged in 13-month-old cKO mice (Supplementary Figure S5).

In order to determine the effects of astroglia-specific *Lsr* deletion on brain lipid homeostasis, RT-qPCR was performed, using primers for cholesterol metabolism-related enzymes and transporters on the same samples used to verify *Lsr* excision (Figure 1B). In the hippocampus of 13-month-old cKO mice, where all Lsr subunits were downregulated by half, both *abca1* (0.761 ± 0.051-fold, *p* = 0.025) and *srebp1* (0.069 ± 0.080-fold, *p* = 0.047) were significantly downregulated as compared to WT mice. This suggests that in the hippocampus, the *Lsr* excision from astroglial cells disturbed cholesterol metabolism, thus affecting both lipogenesis and cholesterol efflux. Although there was a tendency for decreased *abca1* and *cyp46a1* expression in the olfactory bulb, this did not reach statistical significance (Figure 1Bb).

### 2.2. Activity and Anxiety in Astroglial Lsr cKO Mice

**Figure 2:**
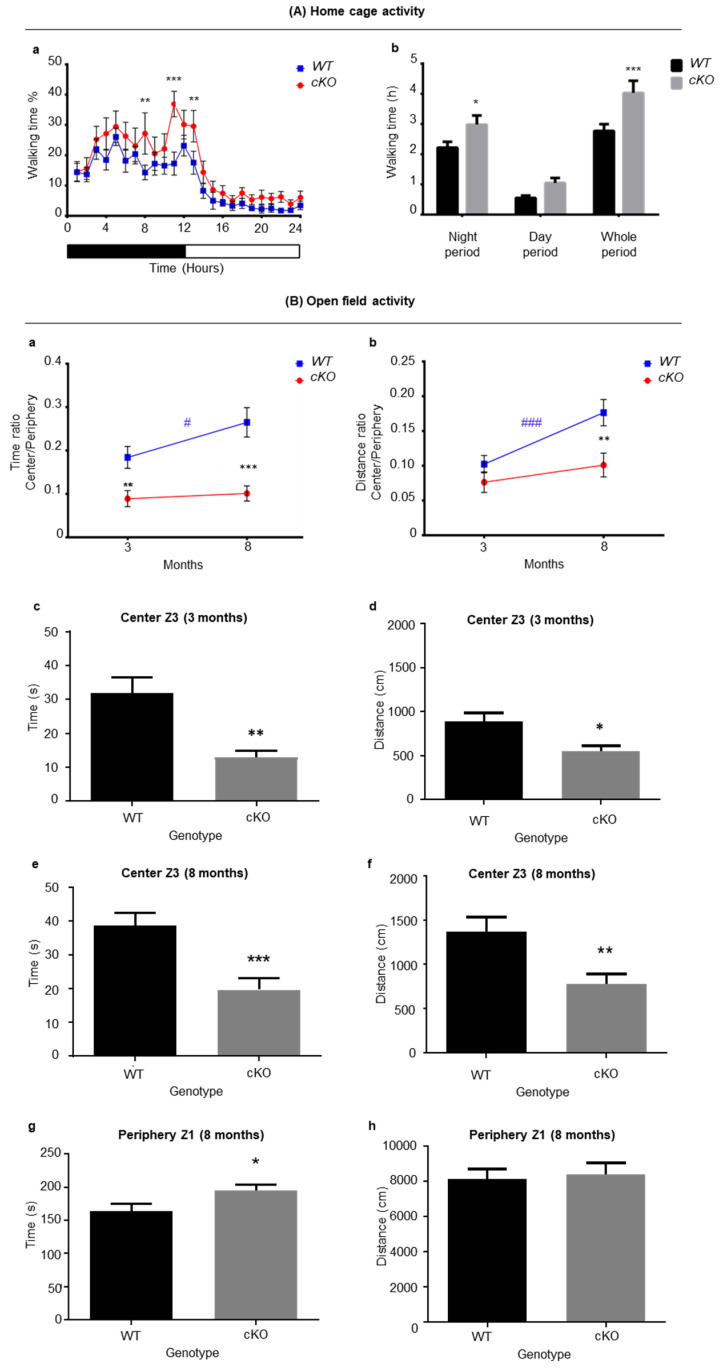
Activity assessment of astroglial Lsr cKO mice. (**A**) Home cage activity in WT (*n* = 18) and cKO (n = 16) mice. *(***a**) Hourly measurements of walking time expressed as % over a 24 h period. (**b**) Sum of walking time in hours (h) during dark cycle, light cycle, and the sum of both cycles (whole period). (**B**) Open field test for WT (n = 20) and cKO (*n* =18) mice. (**a**) Time ratio spent in center (Z3) over that of periphery (Z1). (**b**) Distance ratio traveled in center (Z3) over that of periphery (Z1). (**c**) Time spent in Z3 for 3-month-old mice. (**d**) Distance traveled in Z3 for 3-month-old mice. (**e**) Time spent in Z3 for 8-month-old mice. (**f**) Distance traveled in Z3 for 8-month-old mice. (**g**) Time spent in Z1 for 8-month-old mice. (**h**) Distance traveled in Z1 for older 8-month-old mice. Statistical significance between WT vs. cKO mice is shown as: * *p* ≤ 0.05, ** *p* ≤ 0.01, *** *p* ≤ 0.001; comparison of age (3 months and 8 months) is indicated as: # *p* ≤ 0.05, ### *p* ≤ 0.001.

Home cage activity was assessed on 6–7-month-old animals. The exploratory activity of cKO mice was greater than that of WT mice, with a significant difference between genotypes (Figure 2A, *P <* 0.0001), where cKO mice walked for longer periods of time over the course of 24 h (4.029 ± 0.402 h for cKO mice and 2.768 ± 0.226 h for WT mice, *p* = 0.0004, Figure 2Ab). This was most likely due to increased dark cycle exploration (2.217 ± 0.195 h for WT mice and 2.982 ± 0.300 h for cKO mice, *p* = 0.028, Figure 2Ab). Interestingly, the difference between the distance traveled by cKO and WT mice was insignificant during the dark cycle (Supplementary Figure S6). Indeed, cKO mice (1.208 ± 0.063 cm/s) were generally slower than WT mice (1.359 ± 0.112 cm/s) during the dark cycle, (*p* = 0.017). The cKO mice did not exhibit motor coordination or equilibrium problems after careful observation (stumbling/falling events), thus ruling out the possibility of motor problems. When fine movements, such as scratching and grooming, were measured, cKO mice showed higher levels of such stationary activities (1.874 ± 0.149 h) than WT mice (1.389 ± 0.088 h, *p* = 0.007) over a 24 h period. Closer inspection showed that, in their home cage environment, cKO mice were more active during the last 4 h of the dark cycle (Figure 2Aa), thus exhibiting longer periods of walking time and stationary activities.

The open field test was conducted when mice were 3 and 8 months old. At 3 months of age, WT mice spent more time in the center zone (Z3) than cKO mice, as shown by a significantly higher center/periphery ratio (Z3/Z1) in WT animals (0.184 ± 0.035) vs. cKO (0.089 ± 0.019, *p* = 0.0095, Figure 2Ba,Bc). In addition, data analysis revealed that cKO mice traveled shorter distances in the central zone Z3 when compared to WT mice (median difference of 387.9, *p* = 0.017, Figure 2Bd).

Data showed that at 8 months of age, WT mice actually improved with time (ratio Z3/Z1: 0.184 to 0.265, *p* = 0.02). On the other hand, in cKO mice the ratio of time Z3/Z1 was left nearly unchanged between 3- (0.089 ± 0.019) and 8-month-old mice (0.101 ± 0.018, *p* = 0.74, Figure 2Ba). Thus, the difference between cKO and WT mice was greater at 8 months of age, and the ratio of time spent in the center over the periphery (Z3/Z1) increased significantly for WT (0.265 ± 0.034) compared to cKO (0.101 ± 0.018, *p* < 0.0001, Figure 2Ba) mice. The cKO mice spent more time (194 s) in the peripheral zone Z1 than WT mice (163 s, *p* = 0.038, Figure 2Bg). However, both cKO and WT mice traveled the same distance in Z1 (mean of 8131 cm for WT mice and 8397 cm for cKO mice, Figure 2Bh). This indicates that cKO mice remained in Z1 for longer periods of time. When comparing the time ratio Z3/Z1 in both genotypes with age, a highly significant genotype effect (*p* < 0.0001) was observed. Therefore, in a novel environment, cKO mice tended to stay at the periphery for longer periods of time when compared to WT mice, which may reflect thigmotaxis. This suggested that anxiety, or a lower motivation to explore, reflects apathy in cKO mice. The free exploratory paradigm test was next performed in order to assess mouse anxiety. No significant differences between cKO and WT mice were observed. Both groups spent similar times in new environments (Supplementary Figure S7), indicating that cKO and WT mice exhibit a similar level of anxiety. This supports the hypothesis that the different behavior of cKO mice in the open field is more likely due to apathy rather than anxiety.

### 2.3. Olfaction in Astroglial Lsr cKO Mice

**Figure 3:**
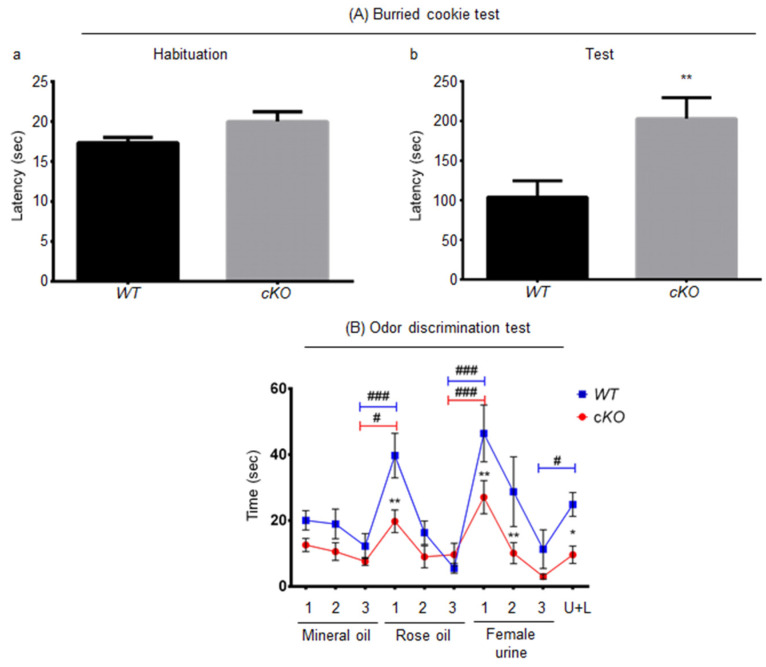
Olfactory assessment in cKO mice. (**A**) Buried cookie test results for WT (*n* = 20) and cKO (*n* = 18) mice. (**a**) Time taken to take and eat the visible cookie in habituation phase. (**b**) Time taken to find buried cookie. (**B**) Habituation/cross-habituation test results for WT (*n* = 10) and cKO (*n* = 10) mice. This test consists of a 5 min habituation step to the tea ball (no odor), then three 1 min habituation sessions to mineral oil, followed by three 2 min habituation sessions to rose oil, and by three 2 min habituation sessions to female urine. The test then ended with a 2 min odor discrimination step to female urine containing 1% lemon oil (U + L). Statistical significance when comparing a single point between cKO vs. WT mice is represented as: * *p* ≤ 0.05, ** *p* ≤ 0.01. Statistical significance when comparing different sessions for same genotype are indicated as: # *p* ≤ 0.05, ### *p* ≤ 0.001.

The buried cookie test was performed when mice were 5 months old. During the habituation phase, in which the cookie is presented visibly to the animal, there was no significant difference between the cKO and WT mice in the time it took to notice and eat the food (Figure 3Aa). However, during the test phase, cKO mice took a 2-fold longer time period to find the buried cookie (203 ± 26.44 s) when compared to WT mice (Figure 3Ab, 104 ± 20.52 s, *p* = 0.0086). In order to verify that this was not a problem linked to satiety or energy homeostasis, body mass (Supplementary Figure S3) and fasting blood glucose levels (Supplementary Figure S8) were measured. There was no significant difference between the two groups, suggesting that the decreased ability to find the buried food in cKO mice was most likely due to olfactory deficits.

To further explore this, odor discrimination was assessed in 10-month-old mice using the habituation/cross-habituation test. There was a significant difference between cKO and WT mice (*p* = 0.0198). The cKO and WT mice responded differently to the sequence at which odors were introduced, with a clear interaction between genotype and time (*p* = 0.0213). When comparing the exploration time of the third introduction of mineral oil and the first introduction of rose oil, WT mice and cKO mice were both able to distinguish the introduction of the new rose odor and discriminate mineral and rose oils, by spending more time sniffing the new odor source (12.39 ± 3.4 s for the third introduction of mineral oil and 38.76 ± 6.11 s for the first introduction of rose odor, *p* < 0.0001, for WT mice; 7.62 ± 1.22 s for the third introduction of mineral oil and 19.77 ± 3.43 s for the first introduction of rose odor, *p* = 0.016, for cKO mice; Figure 3B). However, during the first introduction of rose oil, WT mice showed about twice the interest in the rose odor (38.76 ± 6.11 s) when compared to cKO mice (19.77 ± 3.43 s, *p* = 0.002). To determine whether the mice were habituated to rose oil, interest after the first (cited above) vs. third introduction of rose odor was calculated. Both WT (6.12 ± 1.43 s, *p* < 0.001) and cKO (9.67 ± 3.43 s, *p* = 0.04) mice exhibited decreased interest, reflecting habituation, thus demonstrating that both mouse strains were able to memorize and recognize this specific odor. However, WT mice learned faster (Figure 3B). Both cKO (third introduction of rose odor vs. first introduction of female urine odor, 27.08 ± 5.02 s, *p* = 0.0004) and WT (third introduction of rose odor vs. first introduction of female urine odor, 44.10 ± 8.05 s, *p* < 0.0001) mice were able to discriminate the odors of female urine and rose oil. Although WT mice spent 17 s longer than cKO mice exploring the urine odor (*p* = 0.0056), both were habituated to female urine odor when comparing the first presentation to the third one (cKO mice: 3.02 ± 0.79 s, *p* < 0.0001; WT mice: 10.44 ± 5.33 s, *p* < 0.0001; for the third presentation of female urine odor). Interestingly, cKO mice were unable to discriminate the odor of 1% lemon oil added to female urine (U + L) (third presentation of female urine odor vs. U + L, 9.65 ± 2.65 s, *p* = 0.1859), unlike WT mice (third presentation of female urine odor vs. U + L, 24.89 ± 3.25 s, *p* = 0.011). In addition, WT mice explored the female urine with 1% lemon oil 15 s longer than cKO mice (*p* = 0.0175). In conclusion, cKO mice were able to smell and discriminate odors from very different sources, but showed less ability to discriminate subtle odor changes, such as 1% lemon oil in urine, and spent less time sniffing new odors, suggesting a lack of interest or motivation.

### 2.4. Memory in Astroglial Lsr cKO Mice

**Figure 4:**
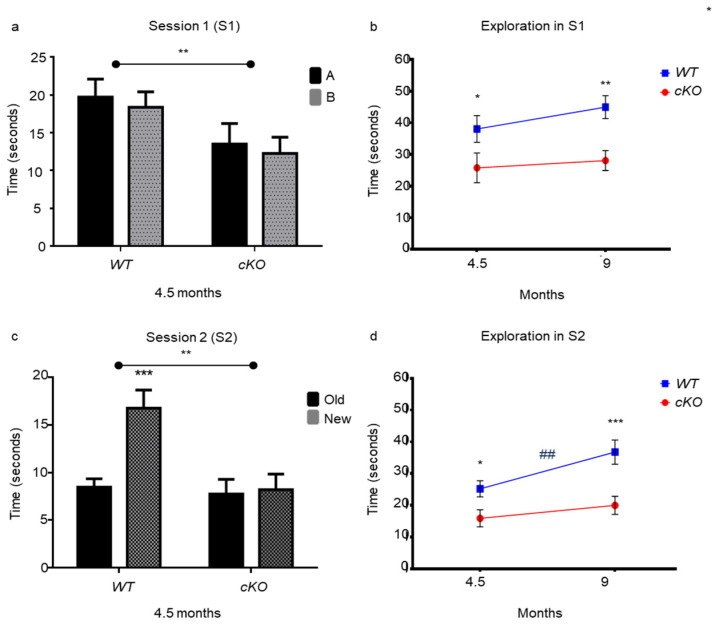
Vision and visual memory assessment of WT (*n* = 20) and cKO (*n* = 18) mice using object recognition test. (**a**) Session 1 (S1): exploration time for the same set of objects at position A and position B. (**b**) Total exploration time in S1 at 4.5 and 9 months of age. (**c**) Session 2 (S2): exploration time of an old object used in S1 and a novel object, which were positioned randomly in the apparatus. (**d**) Total exploration time in S2, in seconds, at 4.5 and 9 months of age. Statistical significance is shown as: * *p* ≤ 0.05, ** *p* ≤ 0.01, *** *p* ≤ 0.001. ## *p* ≤ 0.01.

During the first session (S1) of the object recognition test, 4.5-month-old cKO and WT mice spent almost the same time exploring objects A and B, which are sets of similar objects (WT mice: 19.72 ± 2.36 s and 18.35 ± 2.04 s for the objects A and B, respectively; cKO mice: 13.51 ± 2.70 s for object A and 12.25 ± 2.14 s for object B) (Figure 4a). However, there was a clear genotype effect, since cKO mice spent less total time exploring than WT mice during the first session (Figure 4b, *p* = 0.03). Nevertheless, the object effect was insignificant for both cKO and WT mice, with no genotype–object interaction, indicating that cKO mice had no visual problems in localizing the object, but showed less interest than WT mice to this inanimate stimulus. At both 4.5 (38.08 ± 4.22 s for WT mice and 25.76 ± 4.69 s for cKO mice, *p* = 0.03) and 9 months of age (44.98 ± 3.60 s for WT mice and 28.07 ± 3.15 s for cKO mice, *p* = 0.003), there was a clear genotype effect (*p* = 0.001), but no age effect and no genotype–age interaction (Figure 4b), suggesting that the behavioral profile observed was age-independent.

During the second session (S2), WT mice explored the new object two times longer as compared to the old “familiar” object (old vs. new, 8.45 ± 0.89 s vs. 16.77 ± 1.92 s, *p* = 0.0002). On the other hand, cKO mice explored both new and old objects for the same length of time (old vs. new, 7.75 ± 1.52 s vs. 8.19 ± 1.65 s, Figure 4c). This indicated that 4.5-month-old cKO mice were either unable to discriminate the new object by its form and color, suggesting low visual abilities, or were unable to recall having previously seen the old object before the presentation of the new object (Figure 4c). In addition, cKO mice (15.94 ± 2.68 s) spent less time exploring both objects than WT mice (25.22 ± 2.53 s, *p* = 0.0165, Figure 4d), which would suggest apathy behavior in cKO mice.

**Figure 5:**
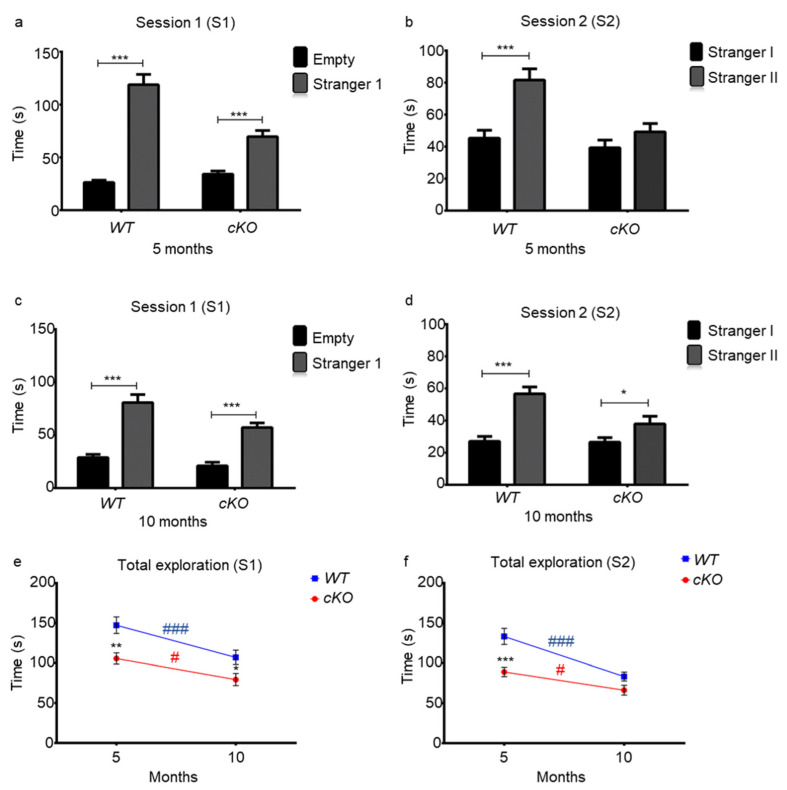
Sociability and social novelty assessment of WT (*n* = 20) and cKO (*n* = 18) mice. 5- (**a**,**b**) and 10- (**c**,**d**) month-old mice. (**a**,**c**) Session 1 (S1): exploration time (s) of stranger 1 vs. empty cup. (**b**,**e**) Session 2: exploration time (s) of stranger 1 used in S1 and a novel stranger. (**e**) Total exploration time in S1, in seconds, at 5 and 10 months of age. (**f**) Total exploration time (s) in S2 at 5 and 10 months of age. Statistical significance between cKO and WT mice at a certain time point is represented as: * *p* ≤ 0.05, ** *p* ≤ 0.01, *** *p* ≤ 0.001. Statistical significance with age in cKO and WT mice is represented as: # *p* ≤ 0.05, ### *p* ≤ 0.001.

The sociability of the two groups was tested at 5 and 10 months of age using the three-chamber sociability and novelty test. During the first session (S1), both 5-month-old cKO (stranger 1 vs. empty cage, 69.65 ± 5.84 s vs. 34.22 ± 3.004 s, *p* = 0.0002) and WT (stranger 1 vs. empty cage, 118.85 ± 9.80 s vs. 26.35 ± 2.17 s, *p* < 0.0001) mice spent more time exploring stranger 1 than the empty cage (Figure 5a). However, cKO mice spent about half the time (69.66 ± 5.84 s) exploring stranger 1 when compared to WT mice (118.86 ± 9.8 s, *p* < 0.0001). There was a genotype effect (*p* = 0.0012), a subject effect (*p* < 0.0001), and a genotype–subject interaction (*p* < 0.0001). These results indicate that cKO mice were less social and less motivated to explore stranger 1, when compared to WT mice.

During S2, when a second novel individual was introduced, WT mice spent more than twice the time exploring stranger 2 when compared to stranger 1 (81.56 ± 6.99 s for stranger 2 and 45.29 ± 4.98 s for stranger 1, *p* < 0.0001). On the other hand, cKO mice spent nearly the same time exploring strangers 1 and 2 (49.22 ± 516 s for stranger 2 and 39.27 ± 4.74 s for stranger 1, *p* = 0.224, Figure 5b). Statistical analyses revealed a significant genotype effect (*p* = 0.001), novelty effect (*p* < 0.0001), and genotype–novelty interaction (*p* = 0.0215). Therefore, cKO mice were unable to discriminate the old from the new individual, indicating a deficit in social memory. As odor trace is the main parameter in individual recognition in rodents, the observed phenotype might be due to olfactory deficits, consistent with the poor performances observed with the olfactory tests described above.

This test was repeated for both groups at 10 months of age. Both cKO mice (stranger 1 vs. empty cage, 56.08 ± 6.27 s vs. 23.26 ± 3.61 s, *p* < 0.0001) and WT mice (stranger 1 vs. empty cage, 78.42 ± 7.78 s vs. 28.56 ± 3.19 s, *p* < 0.0001) were attracted more to stranger 1 than the inanimate object (Figure 5c). During S1, there was a genotype effect (*p* = 0.0025), a subject effect (*p* < 0.0001), but no longer a genotype–object interaction (*p* = 0.122), as was observed when the groups were younger.

During S2, there was a strong genotype effect (*p* = 0.007) and a significant genotype–object interaction (*p* = 0.01). Although both cKO and WT mice spent more time exploring stranger 2 than stranger 1, WT mice explored stranger 2 for a 2.11-fold longer time period than stranger 1 (*p* < 0.0001, Figure 5d). On the other hand, cKO mice showed only 1.4-fold greater interest in stranger 2 compared to stranger 1 (*p* = 0.032, Figure 5d). This indicates that, although cKO mice were able to discriminate and retain memory of stranger 1, it was to a lesser extent as compared to WT mice, which could indicate apathy and lack of interest in social interactions.

Indeed, WT mice exhibited greater interest to congeners than cKO mice. At 5 months of age, the total exploration time of WT mice was 1.38-fold greater than that of cKO mice (Figure 5e). This may reflect a lack of social interest in the cKO mice. With age, the total exploration time decreased significantly in cKO and WT mice in both S1 and S2 (Figure 5e,f). In S1, there was a highly significant time effect (*p* < 0.0001), and a genotype effect (*p* = 0.002), but no genotype–time interaction (Figure 5e). During S2, there was a clear age effect (*p* < 0.0001), and genotype effect (*p* = 0.0003), and a tendency for genotype–age interaction (*p* = 0.068). This indicated a decrease in sociability and willingness to explore with age in both groups.

**Figure 6:**
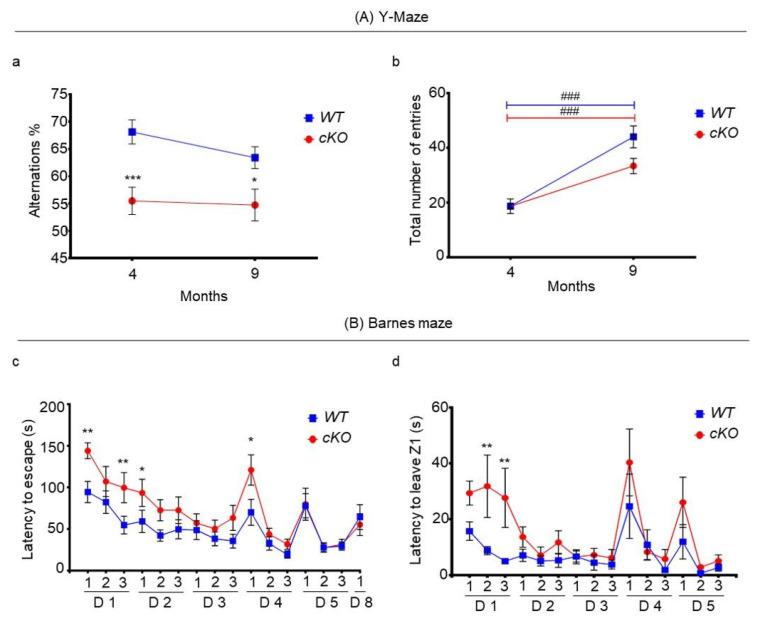
Short- and long-term memory assessment. (**A**) Y-maze for WT (*n* = 20) and cKO (*n* = 18) mice. (**a**) Alternation (%) at the ages of 4 and 9 months. (**b**) Total number of entries at the ages of 4 and 9 months. (**B**) Barnes maze for WT (*n* = 15) and cKO (*n* = 15) mice. (**c**) The time taken to find the escape chamber, measured 3 times per day for 5 consecutive days. On day 8, one single test session was performed to assess long-term memory. (**d**) Time taken to leave departure zone (Z1), measured 3 times per day for 5 consecutive days. Statistical significance between cKO and WT mice at different time points is represented as: * *p* ≤ 0.05, ** *p* ≤ 0.01, *** *p* ≤ 0.001. Statistical significance with time in cKO or WT mice is represented as: ### *p* ≤ 0.001.

To study working memory, 4-month-old animals were tested for spontaneous alternations in the Y-maze, a test to assess spatial short-term memory. The percentage of proper alternations was 68.14 ± 2.2% for WT mice, and 55.5 ± 2.47% for cKO mice (*p* = 0.0005, Figure 6Aa). At this age, both groups had an equal total number of entries: 19 ± 1 for WT mice and 19 ± 2 for cKO mice (Figure 6Ab). Since there were no significant differences in distance and velocity between cKO and WT mice (Supplementary Figure S9A), these results indicate that cKO mice, from a young age, possess short-term memory problems, as they show a decreased ability to recall explored vs. unexplored arms.

At 9 months of age, the percentage of alternations slightly decreased in WT mice to 63.17 ± 2%, whereas it remained fairly constant, but still lower, in cKO mice (54.75 ± 2.88%, *p* = 0.0165, Figure 6Aa). In addition, the measurement of the total number of entries revealed that cKO mice tended to enter 10 ± 5 less arms than WT mice (*p* = 0.0512, Figure 6b). Again, there was no significant differences in velocity and distance between both groups (Supplementary Figure S9B); therefore, the difference in arm entries might reflect a lack of willingness to explore in cKO mice. Regarding percentage alternation, there was a clear genotype effect (*p* < 0.0001), but neither an age effect, nor a genotype–age interaction. This indicates that cKO mice exhibit short-term memory deficits that were not modified with age. However, a higher number of entries with age was observed in both cKO and WT mice (*p* < 0.0001, Figure 6Ab), where there was a tendency for a genotype effect (*p* = 0.085), and a genotype–age interaction (*p* = 0.052). This suggests that both groups may have become more experienced with time due to frequent handling and behavioral testing. Nevertheless, cKO mice tended to explore less arms than WT mice, possibly reflecting a lack of motivation due to apathy.

To assess long-term memory and learning, 11-month-old animals were tested in a modified Barnes maze three times per day for five consecutive days. The time taken to escape scores gradually decreased over the training period (Figure 6Ba) in both groups of mice. There was a clear time effect (*p* < 0.0001), indicating that spatial learning was successfully achieved. When comparing trial 1 on day 1 with trial 3 on day 5, the time to find the chamber decreased by 112.8 s in cKO mice (day 1 trial 1: 144.1 s; day 5 trial 3: 31.3 s; mean difference: 112.8 s, *p* < 0.0001), and 64.8 s in WT mice (day 1 trial 1: 94.4 s; day 5 trial 3: 29.6 s; mean difference: 64.8 s, *p* = 0.0002), demonstrating an ability to learn for both cKO and WT mice. However, there was also a genotype effect (*p* < 0.0001), where on day 1 (trials 1 and 3) and day 2 (trial 1), cKO mice spent significantly more time locating the escape chamber. In trial 1 of day 1, cKO mice spent on average 50 s longer than WT mice to find the escape chamber (WT mice vs. cKO mice: 94.4 ± 12.70 vs. 144.1 ± 9.6 s, *p* = 0.0044). Interestingly, cKO mice on day 1 tended to take longer than WT mice to leave the departure zone Z1 (WT mice vs. cKO mice: 15.7 ± 3.30 vs. 29.3 ± 4.3 s, *p* = 0.062) in trial 1; this difference became statistically significant in the two other trials of day 1 (Figure 6Bb). In trial 1 on day 4, where the subjects were directly placed in Z1 without prior habituation to the escape chamber, the time to leave Z1 was not significantly different between cKO (40.3 ± 12.0 s) and WT mice (24.6 ± 11.5 s) (Figure 6Bb). However, cKO mice spent on average 121 ± 18.3 s to find the chamber, whereas WT mice spent 69.9 ± 15.60 s (*p* = 0.0034) (Figure 6Ba). This indicates that cKO mice demonstrated deficits when confronted with novelty that could reflect a lack of cognitive flexibility. Yet, they were able to retain information with repetition, as in subsequent trials. The time taken to escape was not statistically significant (day 4, trials 2 and 3), even when the starting zone was modified (day 5).

After stopping the test for two consecutive days, a trial was performed on day 8 to assess long-term memory. There was no significant difference between cKO and WT mice, where both groups spent similar times to find the chamber, suggesting no difference in long-term memory between these two groups (Figure 6Ba).

### 2.5. Astroglial Activation in cKO Mice

**Figure 7:**
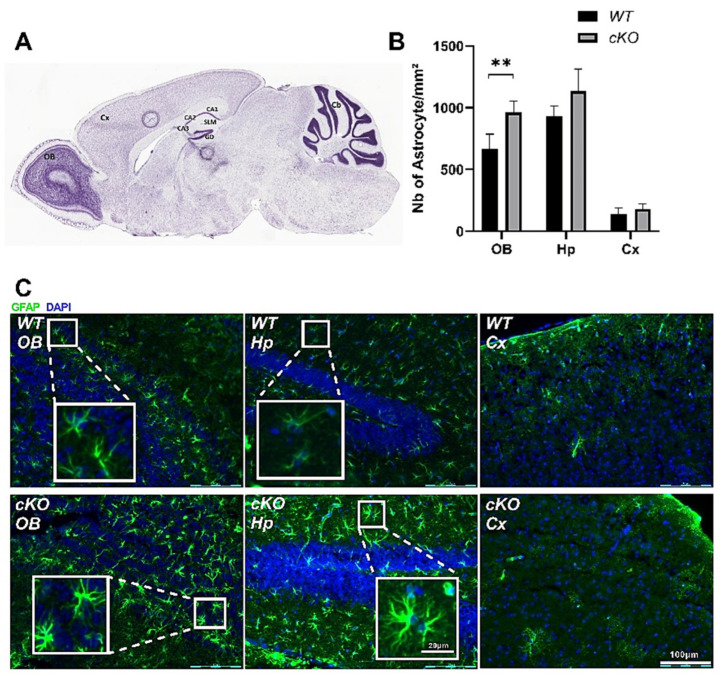
Astrocyte activation in astroglial Lsr in WT (*n* = 5) and cKO (*n* = 5) mice. (**A**) Sagittal brain section corresponding to the regions of interest. (**B**) Number of GFAP+ cells in different brain structures. Sagittal brain sections were stained with glial fibrillary acidic protein (GFAP). The measurements of the number of GFAP+ cells were the result of three sagittal sections analyzed per animal; the mean of all values represents one mouse. (**C**) Representative images from WT and cKO mice with GFAP staining (green) on sagittal sections counterstained with DAPI (nuclear blue staining). Scale bar 100 μm. Each value represents the mean ± SEM (** *p* < 0.01). Abbreviations: OB, olfactory bulb; Cx, cortex; Hp, hippocampus; DG, dentate gyrus; CA, cornu ammonis; SLM, stratum lacunosum-moleculare.

The olfactory bulb (OB), hippocampal (Hp), and cortical (Cx) regions were identified on the sagittal sections according to the mouse brain atlas (Figure 7A). For the quantification of gliosis, glial fibrillary acidic protein (GFAP)-stained areas showing obvious shapes of a cell body were considered as GFAP+ cells. We counted all GFAP+ cells in the whole field of images using three sagittal images per animal, taken from the OB, Hp, or Cx, and compared the results obtained for cKO (*n* = 5) and WT mice (*n* = 5). We observed a significant increase in the number GFAP+ cells in the OB of cKO compared to WT mice (962 ± 40 and 669 ± 52 GFAP+ cells, respectively, *p* = 0.0079). No statistically significant differences were found in the Hp (WT mice: 928 ± 38; cKO mice: 1135 ± 79; *p* = 0.09) or the Cx (WT mice: 141 ± 20; cKO mice: 180 ± 17; *p* = 0.3) (Figure 7B). This increase in GFAP + cells can be seen in the immunohistochemical panel corresponding to the OB of the cKO mice (Figure 7C). In addition, the staining intensity and shape of the cells were different between the cKO and WT sections of the OB. The cells appeared bigger and more ramified in cKO mice. The number of branches were quantified, and the results showed a significant increase in cKO mice (7.3 ± 0.33) compared to WT (4.25 ± 0.17, *p* < 0.001) (Supplementary Figure S10). Moreover, GFAP+ cells in cKO mice exhibited larger cell bodies and cytoplasmic extensions in both the OB and the Hp. No major changes were noticed in the Cx. Similar images were observed in the Hp even when the total number of GFAP+ cells was unchanged. Altogether, our findings suggest astroglial activation in cKO mice.

## 3. Discussion

The induction of CRE enzyme activity by tamoxifen led to the excision of the *Lsr* gene in GLAST-expressing cells. In 3-month-old cKO mice, we detected a significant reduction in *Lsr* expression in both the Hp and the OB (Supplementary Figure S4). In older animals, after the battery of behavioral tests, we observed that *Lsr* expression was still significantly reduced in the Hp of cKO mice (Figure 1Aa), whereas the OB only exhibited reductions in some isoforms (Figure 1Ab), or even no reduction at all, such as in the CB (Supplementary Figure S5). The discrepancies between brain regions may be due to the various levels of CRE and GLAST in the different regions. Moreover, compensatory mechanisms may occur with time in specific brain regions, leading to an overexpression of *Lsr* in non-glial cells, thereby masking the efficiency of the gene excision. Indeed, even if astroglial cells have been shown to express a major part of the *Lsr* found in the CNS [18], other cells, including neurons, may modulate their expression levels in response to tamoxifen treatment or astroglial *Lsr* depletion.

Nevertheless, specific in vivo suppression of *Lsr* in the astroglial cells led to the perturbation of cholesterol homeostasis in the CNS. Indeed, the significant downregulation of *Lsr* in the Hp was associated with the downregulation of *srebp1*, which is a transcriptional regulation factor of genes responsible for de novo lipogenesis, and *abca1*, which codes for the ABC transporter responsible for cholesterol efflux and extracellular transport of lipoproteins. Therefore, LSR may play a role in modulating cholesterol synthesis and transport through SREBP1 and ABCA1. Indeed, SREBP1 mediates the activation of lipoprotein receptor promoters, such as that for LDL-R [20,21,22]. Further investigation is underway to understand how LSR downregulation is linked to perturbations in cholesterol homeostasis. Based on the genes affected in the cKO mice, as well as our previous studies showing changes in cholesterol homeostasis in *Lsr+/−* mice [13,23] this may involve cholesterol metabolism-related proteins, oxysterol accumulation, and the LXR pathway. Indeed, the fine control of cholesterol trafficking in the brain is essential for proper neuronal function. 

Behavioral analysis revealed several specific traits in cKO mice compared to WT animals. In addition, a series of traits were also correlated to aging in the cKO mice (Supplementary Table S1). In their environment, cKO mice remained active for longer periods of time compared to WT mice during the nocturnal period (Figure 2Aa), particularly at the beginning and at the end of the nocturnal period. This has also been observed in AD mice models, in which mice were found to be hyperactive during the nocturnal phase [24,25,26]. In a novel environment, cKO mice tended to stay in the peripheral zone for longer periods of time when compared to WT mice, which can be a sign of thigmotaxis. Nevertheless, they traveled the same distance at the periphery, which indicates longer immobile periods at the periphery. Together, the observed immobility and thigmotaxis could reflect a form of anxiety. However, the results of the free exploratory paradigm excluded anxiety behavior in cKO mice. Instead, cKO mice exhibited signs of apathy. Interestingly, apathy is also considered to be an early marker for neurodegenerative diseases. In addition, patients with mild cognitive impairment (MCI) exhibiting apathy are at a greater risk of developing AD compared to those with no neuropsychiatric symptoms [27].

The cKO mice were able to visualize objects and visual cues, since they explored the same set of objects for a nearly equal amount time (Figure 4a). Furthermore, they were able to visualize the geometric cues in the Barnes maze to find the escape chamber (Figure 6c). However, they were unable to memorize or discriminate between an old and a new object (Figure 4c). They also showed a lower percentage of alternation in the Y-maze than the controls, indicating a deficit in recalling previously visited arms (Figure 6a). Taken together, these results suggest that visual and working memory were affected in our animals. They were able to navigate and explore their environment as they received sufficient visual sensorial stimulation, and did not suffer from locomotor problems. However, they were unable to retain information sufficiently or properly to achieve a specific task with the same efficiency as WT animals. Visual and working memory deficits are early markers of ongoing neurodegenerative processes in AD [28,29,30]. Impaired visual recognition memory and working memory decline are markers for MCI and its evolution towards AD [31,32]. In our animals, the working memory performance was lower in cKO mice than in WT mice, both in young and older animals; however, while it declined in WT mice, reflecting a normal aging process, it appeared more stable in cKO mice, suggesting cognitive restructuring or neuroplasticity.

With regards to olfaction, cKO mice took twice the time to find the buried cookie as compared to controls. In addition, they spent less time sniffing new odors and were unable to discriminate between subtle odors. Our results indicate that olfactive memory exists in cKO mice, but it is less efficient than in WT mice. This impaired sensorial entry, essential for rodent identification, might have caused cKO mice to engage less in social interactions than WT mice, thus explaining the lower sociability of cKO mice. Interestingly, deficit in olfactory discrimination is an important early diagnostic tool for neurological disorders [33]

In most tests, cKO mice displayed apathetic-like behavior. In addition, 11-month-old cKO mice also showed low cognitive flexibility and a lack of motivation to explore the novel surroundings, which could be overcome by repetitive introduction to the aversive environment of the Barnes maze. Low cognitive flexibility and lack of motivation are two well-documented behaviors found in AD patients [34,35].

The results of this study show that astroglia-specific deletion of *Lsr* led to a series of defects affecting cKO behavior. Some of these behavioral traits are found in neurodegenerative diseases, including MCI and AD [36]. The mechanisms underlying the observed phenotypes following *Lsr* deletion remain to be deciphered. However, ApoE ε4 is a strong AD risk factor [37], and represents a potential link between lipoprotein trafficking, cholesterol homeostasis, and neurodegenerative processes. It has been suggested that poor the loading ability of ApoE ε4 lipoproteins may lead to deficient β-amyloid clearance and cholesterol trafficking in AD patients, leading to synaptic dysfunction, destruction, and neuronal loss, thereby triggering glia cell activation and inflammatory processes [38,39,40,41,42]. Here, we observed an increased number of GFAP+ cells in the olfactory bulb, and changes in astrocyte morphology in the olfactory bulb and the hippocampus, suggesting astroglial activation in these brain regions (Figure 7). Interestingly, both structures are involved in the main behavioral traits identified as dysfunctional in cKO mice, suggesting that astrogliosis could contribute to the observed phenotypes. In addition, qPCR analysis revealed a significant decrease in brain-derived neurotrophic factor (BDNF) in the OB of cKO mice (Supplementary Figure S11). BDNF is a neurotrophic factor with anti-inflammatory and neuroprotective properties [43]. The inherently higher astroglia reactivity associated with *Lsr* depletion in these cells may further exacerbate neurodegenerative events in a process involving inflammatory signals yet to be deciphered. A link between cholesterol perturbation and inflammation has been shown in Niemann–Pick type C disease, a lysosomal storage disorder in which cholesterol accumulation leads to microglial activation and inflammatory processes, leading to neuronal death in the olfactory bulb, causing olfactory dysfunction [44]. It is possible that a similar mechanism may occur in the OB of cKO mice, where the deletion of *Lsr* in astroglial cells causes cholesterol perturbation, astrocyte proliferation and activation, inflammatory processes, and, ultimately, olfactory dysfunction. Additional studies are underway to confirm this hypothesis.

We postulate that astroglial LSR may play a role in the feedback control of cholesterol synthesis, limiting circulating cholesterol in brain extracellular fluid, thus maintaining cholesterol homeostasis. LSR deficiency might lead to the dysregulation of cholesterol efflux from astrocytes. 

Taken together, our previous studies and current observations demonstrate LSR as a pivotal element in astrocytes involved in normal brain aging. We therefore propose that LSR represents a novel pathway to study the link between cholesterol trafficking, astrogliosis, neuroinflammation, and neurodegenerative processes.

## 4. Materials and Methods

### 4.1. Animals

Animal studies were conducted in accordance with the European Communities Council Directive (EU 2010/63) for the use and care of laboratory animals. All experimental procedures were carried out in accordance with the ethical committee CELMEA N°066 (authorization number: APAFIS #12079-201711081110404). Animals were housed in certified animal facilities (#B54-547-24) of the Bioavailability–Bioactivity (Bio-DA) platform, with a mean temperature of 21–22 °C and relative humidity of 50 ± 20%, and provided a standard chow diet (Envigo Teklad, Gannat, France) and water ad libitum. A reverse 12 h light (midnight-noon)/dark (noon-midnight) cycle was used to allow for behavioral tests during the active nocturnal phase. Floxed *lsrfl/fl* mice were generated and obtained from the Mouse Clinical Institute (ICS, Illkirch, France). Mice expressing Cre-recombinase under an inducible GLAST promoter, Tg(Slc1a3-cre/ERT2)45-72Fwp, were kindly provided by Prof Franck Pfrieger. To generate conditional *Lsr* knockout male mice (cKO, *n* = 18), Tg(Slc1a3-cre/ERT2)45–72Fwp mice were crossed with *lsrfl/fl* mice; outbred males and females carrying both the *Cre* allele and the floxed *Lsr* allele were then crossed with homozygous *lsrfl/fl* mice to obtain mice with the *Cre* allele and homozygous for floxed *Lsr* (Supplementary Figure S1). Wild-type male mice (WT, *n* = 20) with the same genetic background (C57BL/6J) were used as controls (Charles River, Saint Germain Nuelles, France).

### 4.2. Tamoxifen (TAM)-Induced Activation of CreERT2

TAM (Sigma, St Louis, MO, USA) was dissolved in a 9:1 (*v*/*v*) sunflower oil (Sigma) and ethanol (Carlo Erba Reagents, Val de Reuil, France) at a concentration of 15 mg/mL at 37 °C, and then sterile-filtered and stored for up to 7 days at 4 °C in the dark. A 23 G needle tuberculin syringe (Henke Sass Wolf, Tuttlingen, Germany) was used for intraperitoneal injections. At the age of 8 weeks, all mice were injected for 5 consecutive days (every 24 h) with 150 µg of TAM per g of body weight [19]. Each mouse was randomly chosen from a different litter to avoid any litter-specific bias.

### 4.3. Behavioral Tests

Two weeks after TAM induction, behavioral tests were performed over an 8 months period using the same mice throughout the entire study (Supplementary Figure S2). The open field test (OFT) was performed on 3- and 8-month-old mice, object recognition test (ORT) on 4.5- and 9-month-old mice, the Y-maze on 4- and 9-month-old mice, buried cookie test on 5-month-old mice, three-chamber sociability and social novelty test (C3C) on 5- and 10-month-old mice, home cage activity test on 6-month-old mice, color discrimination on 10-month-old mice, free exploration on 10-month-old mice, Barnes maze on 11-month-old mice. All the mentioned tests were performed 1 h after the beginning of the dark cycle under red light.

#### 4.3.1. Activity and Anxiety

##### Home Cage Activity Test

Home cage activity test was performed at the age of 6-7 months on cKO mice (n = 16) and WT mice (n = 18). General activity, including walking time and fine movements, was measured by monitoring mice, using the Promethion High-Definition Behavioral Phenotyping System (Sable Instruments, Inc, Las Vegas, NV, USA), over a period of 24 h. The mice were left in their own individual cages (308 L × 115 W × 120 H cm), with food and water ad libitum. Instrument setup and data acquisition was conducted using MetaScreen software version 2.2.18.0, and the raw data obtained were then processed via ExpeData version 1.8.4 using an analysis script for data transformation, following Sable’s system guidelines. Ambulatory and voluntary activities and animal positions were monitored simultaneously by collecting the data using the XYZ beam arrays with a beam spacing of 0.25 cm.

##### Open Field Test

The open field test was performed twice at the ages of 3 and 8 months on cKO mice (*n* = 18) and WT mice (*n* = 20). The test apparatus consisted of a large circle-shaped frame (diameter, 80 cm × height, 60 cm), virtually divided into three different zones using the SMART software (Bioseb, Vitrolles, France), as follows: an external zone Z1 (40 cm radius), an intermediate zone Z2 (26.7 cm radius), and a central zone Z3 (13.3 cm radius). The apparatus was illuminated on opposite sides with two lamps, where the center (Z3) was illuminated at 120 lux and the periphery with dimmer light. Each animal was placed individually on the same side of Z1, facing the wall, and allowed to explore freely for 5 min. After each trial, the test arena was cleaned thoroughly with 5% (*v*/*v*) ethanol solution [45]. The following parameters were recorded: time spent in each zone, total distance, average velocity, as well as number of entries into each zone.

##### Free Exploratory Paradigm Test

The free exploratory paradigm test was performed at the age of 10 months with 15 cKO mice and 15 WT mice. We followed the same protocol described previously [46,47]. Briefly, the apparatus was a polyvinyl chloride box (60 L × 42 W × 22 H cm) covered with Plexiglas and subdivided into six equal square units interconnected by small holes. The box can also be divided into two by means of three temporary partitions. Twenty-four hours before testing, each animal was randomly placed into one half of the apparatus, which constituted the “familiar” compartment. During this time, the other compartment remained inaccessible to animals by placing partitions. The floor of the familiar compartment was covered with fresh sawdust, and the animals had ad libitum access to food and water. At the start of the test, the animals were exposed to both familiar and novel environments by removing the partitions without themselves being removed from the box. Then, the behavior of animals was recorded under red light for 5 min, using a video camera in infrared mode.

#### 4.3.2. Olfaction

##### Buried Cookie Test

The buried cookie test was performed at the age of 5 months with 18 cKO mice and 20 WT mice [48,49]. On the first two days, mice were habituated to the cookie (Honey pops, Kellogg’s, Limoges, France), where each mouse was given a cookie per day, and the time taken to approach and start eating the cookie was monitored. On day three, the mice were fasted 6–7 h before the test time, with free access to water. The test was carried out in the housing room of the tested mice, and in their own cages. A barrier was put in the middle of the cage, and the cookie was buried 1 cm under the litter on the opposite side of the cage. The test was filmed using a video camera in night mode, the barrier was removed and the time taken to find and start eating the cookie was measured.

##### Habituation/Cross-Habituation Test

The habituation/cross-habituation test was performed when cKO (*n* = 10) and WT (*n* = 10) mice were 10 months old to assess odor discrimination performances. This test consisted of three habituation trials, each for 1 min, to a tea ball containing mineral oil (Sigma-Aldrich), separated by a 1 min interval each time. Odor memorization would be identified by a reduction in time spent sniffing the tea ball containing a similar odor over the three trials. Then the test was continued with three 2 min habituation trials with rose oil, separated by 1 min intervals, in order to measure the ability of the mice to perform new vs. old odor discrimination. This was followed by three 2 min habituation trials with female urine, separated by 1 min intervals, in order to identify social odor discrimination. The test then ended with a 2 min odor discrimination step using female urine containing 1% lemon oil, in order to identify fine odor discrimination abilities [48,49,50].

#### 4.3.3. Memory

##### Object Recognition Test

The object recognition test was performed twice on 4.5- and 9-month-old cKO (*n* = 18) and WT mice (*n* = 20). It was conducted to assess vision and visual memory, and was carried out in a dimly illuminated room (25 lux), in a square opaque plastic box (30 L x 30 W × 26 H cm). Two different sets of objects were used, either colorful plastic blocks (Lego, Billund, Denmark) or litter-filled Falcon tubes with red caps. The test was separated into three steps, a habituation step, a familiarization session, and a visual memory and novelty session. At first, mice could habituate to the empty arena and freely investigate. In the second step, mice were left to explore two similar objects, i.e., either two Lego blocks or two litter-filled Falcons, where the objects were placed in two opposite corners of the arena; namely positions A and B (session 1, S1). After 1 h, in the visual memory session, the former object, either a Lego block or a Falcon tube, and a new object, either a Falcon tube or a Lego block, were placed in the apparatus randomly, either in position A or B (session 2, S2). The 3-points tracking mode of the SMART video-tracking system (Bioseb, Vitrolles, France) was used to monitor the object exploration using nose-point detection. In S1 and S2, a mouse was considered to be exploring an object when its nose was directed toward, and not farther than 2 cm away from, the object [51].

##### Three-Chamber Sociability and Social Novelty Test

The three-chamber sociability and social novelty test was performed twice, at 5 and 10 months of age on cKO mice (*n* = 18) and WT mice (*n* = 20) to assess sociability and social memory. The social approach apparatus was an open box made of acrylic (63 L × 42 W ×  23 H cm) divided into three chambers with two gray acrylic walls. Dividing walls had retractable doors allowing access into each chamber. The wire cup used to contain the unfamiliar mice was made of cylindrical chrome bars spaced 1 cm apart (height, 10 cm; bottom diameter, 10 cm). The test was conducted in a 65 lux illuminated room. Test mice were placed in the central chamber at the beginning of each 5 min phase. During the habituation phase, each of the two side chambers contained an empty inverted wire cup. During the sociability phase (session 1, S1), an unfamiliar mouse (stranger 1) was enclosed in one of the wire cups in a side chamber. The location of stranger 1 alternated between the two side chambers. During the social novelty phase (session 2, S2), a new unfamiliar mouse (stranger 2), from a different cage than stranger 1, was enclosed in the wire cup that had remained empty during the sociability phase. Exploration of an enclosed mouse or an empty wire cup was defined as when a test mouse oriented toward the cup, with the distance between its nose and the cup being less than 1 cm. The time spent in each chamber, and time spent exploring enclosed mice or empty cups, was recorded from a camera mounted overhead and analyzed afterwards by random order. All mice used as “strangers” were young male mice habituated to being enclosed in inverted wire cups in the three-chamber apparatus for 5 minutes daily on two consecutive days prior to the experiment [52].

##### Y-Maze Test

The Y-maze test was performed on 4- and 9-month-old cKO mice (*n* = 18) and WT mice (*n* = 20) to assess spatial short-term memory. The 3 arms-maze was made of opaque Plexiglas, where each arm was 40 cm long, 16 cm high, and 9 cm wide, and positioned at equal angles. Mice were placed at the end of one arm, namely arm A, before starting the experiment countdown. The series of arm entries were recorded visually, and arm entry was validated when the hind paws of the mouse were completely placed in the arm. Spontaneous alternation behavior was monitored during a 5 min time period. Alternation scores were calculated by analyzing overlapping triplet sets, a sequence of unique visits to the three different arms, reflecting a memorization of the already visited arms. The proper alternation percentage was calculated as the ratio of proper alternation overlapping triplets over the total number of entries, multiplied by 100 [13].

##### Barnes Maze

The Barnes maze was performed when cKO (*n* = 15) and WT mice (*n* = 15) were 11 months old to assess spatial learning abilities and long-term memory. The prototype used was a modified version of the initial maze [53]. It consisted of a circular, 120 cm wide table, with 40 randomly placed 5 cm diameter holes to avoid bias induced by serial search strategies usually shown by mice, and placed 40 cm from the ground [54]. The platform was virtually divided into four equally portioned zones using the SMART video tracker software. There were distant and proximal visual cues placed on the left, right, above, and around the platform; the visual cue ‘’X’’ was positioned in the zone where the only escape chamber was placed. By sprinkling water and using two fans, the platform was made aversive. The test was repeated for 5 uninterrupted days with three consecutive trials per day. Each trial was ended when the mouse found the escape chamber; if the chamber was not found, the test was stopped after 3 min. For the first three days, the mice were placed into the escape chamber for 1 min before the test started, to increase their motivation for finding the escape chamber during the test. Each session started in the zone opposite to the escape zone. On the fourth day, the test was conducted without the 1 min session in the escape chamber, and on the fifth day, the test sessions were launched in another zone to assess cognitive flexibility. To assess long-term memory, two days after the fifth session, a single test session was performed starting in the same launch zone as day 4.

### 4.4. RNA Extraction and Real-Time (RT) Quantitative PCR (qPCR)

At the age of 3 months, six cKO and six WT mice were sacrificed by decapitation after isoflurane anesthesia in order to preserve the integrity of the brain structure. Total RNA was extracted from OB and Hp using the NucleoSpin^®^RNA Plus kit (Macherey-Nagel, Düren, Germany). RNA was reverse transcribed using random primers, dNTP, RNAseout, DTT, and MML-V reverse transcriptase (all from Invitrogen, Cergy Pontoise, France), following the manufacturer’s instructions. The qPCRs were performed in triplicate using Takyon No ROX SYBR MasterMix blue dTTP (Eurogentec, Liège, Belgium) and a Mastercycler Realplex^2^ Real-Time PCR System (Eppendorf, Hamburg, Germany). The cycling program was as follows: 5 min at 95 °C, 40 cycles of 15 s at 95 °C, and 45 s at 60 °C. The qPCR efficiencies were controlled with standard curves. Data were analyzed using the Pfaffl model [55]. This method was used to measure mRNA levels of total *Lsr*, complement component 3 (C3, which plays a central role in complement activation), S100 calcium-binding protein β (S100*β*), an astrocytic marker, and brain-derived neurotrophic factor (BDNF, a member of the neurotrophin family of growth factors with anti-inflammatory properties). Relative expression of transcripts of interest was standardized using the following 3 housekeeping transcripts: eukaryotic elongation factor 2 (*EEF2),* peptidyl-prolyl cis–trans isomerase A (*PPIA),* and eukaryotic translation initiation factor 3 subunit F *(EIF3F*). Primers (Eurogentec) used to amplify transcripts were designed in different exons to avoid the amplification of potential genomic DNA traces (Table 1). Primer specificity was checked using a Basic Local Alignment Search Tool (BLAST) search through the US National Center for Biotechnology Information (Bethesda, MD, USA).

At the age of 13 months, freshly collected tissues from five cKO and five WT mice were conserved in RNAlater (Qiagen, Les Ulis, France) as per the manufacturer’s instructions, and stored at −80 °C until use. Different brain regions were isolated, and total RNA was extracted using TRI reagent (Sigma-Aldrich), according to the manufacturer’s instructions, after homogenization using 23 G needles. RNA quantity and purity were evaluated using the Nanodrop ND-1000 spectrophotometer (Thermo Scientific; Villebon-sur-Yvette, France), and the samples with a 260/280 nm ratio ≥ 1.7 were used for subsequent analyses. Reverse transcription was performed using 1 μg of RNA in a final volume of 20 μL, including 0.5 μL of random primers (3 mg/mL), 1 μL of 10 mM dNTP mix in RNase-free water (all from Invitrogen). After denaturation of RNA samples at 65 °C for 5 min, 4 μL of buffer (5×), 2 μL of 0.1 mM DTT, 1 μL of Superscript II reverse transcriptase, and 1 μL of RNase OUT (all from Invitrogen) were added, and transcribed in an Applied Biosystems 2720 thermal cycler according to the following conditions: 25 °C for 10 min, 42 °C for 50 min, and 70 °C for 15 min. The cDNA from individual animals was used as a template for the PCR array using the PowerUP SYBER Green master mix from Applied Biosystems (Foster City, CA, USA) with the following final concentrations in a 25 μL final volume: 1 × Master Mix, 100 nM forward and reverse primers, 0.4 ng/μL cDNA. The mix was placed in a 7500 Fast Real-Time PCR system (Applied Biosystems). The thermal cycling conditions were an initial 5 min denaturation at 95 °C, followed by 42 cycles of 15 s at 95 °C, 1 min at 60 °C, and a final dissociation step. The primer specificity was determined based on the presence of a single peak in the melting curve. We followed eight target mRNA sequences (total *Lsr*, *Lsr α, Lsr α’*, *Lsr β*, *abca1*, *hmgcr, srebp1*, and *cyp46a1*), and their expression levels were compared to those of three reference sequences (hypoxanthine guanine phosphoribosyl transferase (*Hprt*) [12], phosphoglycerate kinase 1 (*Pgk1*), and transferrin receptor protein 1 (*Tfrc1*) [56]) (Table 1). Lsr primer sequences were selected using the Primer-BLAST Genbank based on *Lsr* gene sequence (NM_017405). Quantitation was performed by the 2^-∆∆Ct method [57]. The obtained results were tested for statistical significance (*p* < 0.05) using the Relative Expression Software Tool 2009 (REST Version 2.0.13). The fold changes of mRNA samples of cKO animals were compared to WT animals.

### 4.5. Blood Glucose Test

Five-month-old mice were fasted for 6 h before the test; water was provided ad libitum. Blood samples were collected from the central tail artery using a sterile 25 G needle. No anesthesia was used at the time of blood sampling to avoid unequal variations between animals and avoid the effects of anesthesia on the blood glucose levels. Mice were maintained immobile in a small retainer during blood collection from the tail. Blood samples were collected by skilled personnel. The ACCU-CHEK Performa glucometer (Roche Diabetes Care France, Meylan, France) and ACCU-CHEK strips (Roche Diabetes Care France) were used to determine plasma glucose levels.

### 4.6. Immunohistochemistry

At the age of 13 months, five cKO and five WT mice were sacrificed by decapitation after isoflurane anesthesia in order to preserve the integrity of the brain structure. Brains were fixed by immersion in 4% PAF (Sigma-Aldrich) in 100 mM phosphate buffer solution (PBS) for 24 h at 4 °C. Brain architecture was cryoprotected in PBS containing 30% sucrose before embedding the whole brains in Optimum Cutting Temperature (OCT) compound (VWR, Fontenay-sous-Bois, France). For histology, 10 µm thick sagittal cryosections were made using a HM550 Cryostat (Microm Microtech, Brignais, France) and collected onto Superfrost+ slides (VWR). Brain tissue sections were permeabilized and blocked using a 0.2% Triton 100 (Sigma-Aldrich) and 30% Cas-Block (Invitrogen) solution prepared in 1X D-PBS (Invitrogen) for about 1 h at room temperature. Sections were then incubated overnight at 4 °C with mouse anti-GFAP primary antibody (MAB360, Chemicon, CA, USA) used at 1:500 dilution in D-PBS containing 0,02% Triton x100 and 3% Casblock. After washing, this was followed by 1 h incubation with goat anti-mouse secondary antibody coupled to Alexa 488 fluorochrome (ref A11001, Thermo Fisher) prepared at 1:500 dilution in D-PBS. Sections were counterstained with DAPI (D9542, Sigma) used at 10 μg/mL, then mounted in Fluoromount-G (Electron Microscopy Sciences, Hatfield, PA, USA), and left at room temperature overnight, protected from light. Slides were then examined using a fluorescence microscope (Leica Microsystems). Image analysis and measurements were performed with Image J.

### 4.7. Statistical Analyses

To verify that the behavioral data obtained followed a Gaussian distribution, we used the Kolmogorov–Smirnov normality test. After verifying that all the data were of Gaussian distribution, student’s *t*-test (two tailed, unpaired) was performed to compare cKO and WT mouse data with one factor (genotype). Two-way analysis of variance (ANOVA) was performed to analyze data containing two different factors (example genotype x time). The numerical and graphical results are presented as mean ± standard error of the mean (SEM). The degree of statistical significance was set at a level of *p* ≤ 0.05 (*), *p* ≤ 0.01 (**), *p* ≤ 0.001 (***). Statistical calculations were carried out using the Statview 4.5 statistical package (Abacus Concept, Int.) and Excel 6.0 (Microsoft, Inc.).

For RT-qPCRs, the statistical data in the boxplot were obtained using REST software tool, where (+) represents the mean value, the middle line represents the median, the lower (Q1) and upper (Q3) lines in the bar represent the 25% and 75% quartile, respectively. While the upper and lower lines represent the observations outside the 9–91 percentile range, data falling outside of the Q1 and Q3 range are plotted as outliers of the data.

## Figures and Tables

**Table 1:** Sequences of RT-qPCR primers used in the study. Forward and reverse primers of the reference genes used: *EEF2, EIF3F,* *Hprt, Pgk1*, *PPIA,* and *Tfrc*. Target isoforms of *Lsr*: total (T), α, α’, and β. Cholesterol metabolism genes: *abca1*, *cyp46a1*, *hmgcr,* and *srebp1*. Inflammatory and neurotrophic genes: *BNDF, C3,* and *S100β*.

Gene	Forward Primer (5′ to 3′)	Reverse Primer (5′ to 3′)
*Abca1*	CAACCCCTGCTTCCGTTATCCAA	GAGAACAGGCGAGACACGATGGAC
*BDNF* *C3* *Cyp46a1*	TGGCCTAACAGTGTTTGCAG AGAGGCAAGTGCTGACCAGT GGCTAAGAAGT TGGTCCTGTTGTAAGA	TGTCAGCTCCACTTAGCCTC ACTGGCTGGAATCTTGATGG GGTGGACATCAGGAACTTCTTGACT
*EEF2* *EIF3F* *Hmgcr*	GTGGTGGACTGTGTGTCTGG CATCAAGGCCTATGTCAGCA CCCCACATTCACTCTTGACGCTCT	CGCTGGAAGGTCTGGTAGAG AGGTCAACTCCAATGCGTTC GCTGGCGGACGCCTGACAT
*Hprt*	TCAGACTGAAGAGCTACTGTAATGATCA	AAAGTTGAGAGATCATCTCCACCAA
*Lsr (total)*	AGTAATACACTCCACTGTCTCCCCAG	CAGGAGAATCACCATCACAGGAA
*Lsr α*	AAGATCTGGATGGGAACAACGAG	CTTCTGAGGTCCTGCCAAGG
*Lsr α’*	AAGATCTGGATGGGAACAACGAG	CAAAGAGCCAATCAAGGACAATG
*Lsr β*	AAGATCTGGATGGGAACAACGAG	CCAGCAGCATAAACAAGGACAAT
*Pgk1*	GAGCCTCACTGTCCAAACTA	CTTTAGCGCCTCCCAAGATA
*PPIA* *Srebpf1* *S100β*	GTCTCCTTCGAGCTGTTTGC GGTCCAGCAGGTCCCAGTTGT AACGAGCTCTCTCACTTCCT	GCGTGTAAAGTCACCACCCT CTGCAGTCTTCACGGTGGCTC AAAGAACTCATGGCAGGCCG
*Tfrc*	GTCTTCTGTTGAAACTTGCCCA	GAAAGGTATCCCTCCAACCACTC

## Data Availability

Data are contained within the article or supplementary material.

## Supplementary Materials

The following are available online at https://www.mdpi.com/article/10.3390/ijms23042049/s1.

## Abbreviations

ABCA1	ATP-binding cassette transporter A1
ABCG1	ATP-binding cassette transporter G1
AD	Alzheimer’s disease; Apo, apolipoprotein
BB	blood brain barrier
BDNF	brain-derived neurotrophic factor
cKO	astroglial *Lsr* knock-out
CNS	central nervous system
C3	complement component 3
Cre	Cre-recombinase
Cx	cortex
Cyp46A1	cytochrome P450 family 46 subfamily A member 1
EIF3F	eukaryotic translation initiation factor 3 subunit F
ERT2	estrogen receptor inducible promoter
fl	flox
EEF2	eukaryotic elongation factor 2
GFAP	glial fibrillary acidic protein
GLAST	glial glutamate transporter
hmgcr	HMG CoA reductase
Hp	hippocampus
LSR	lipolysis-stimulated lipoprotein receptor
MCI	mild cognitive impairment
OB	olfactory bulb
PPIA	peptidyl-prolyl cis–trans isomerase A
RT-qPCR	real-time quantitative PCR
S1, S2	session 1, session 2
S100*β*	S100 calcium-binding protein β
srebp1	sterol regulatory element-binding transcription factor 1
TAM	tamoxifen
WT	wild-type
Z1,2,3	zones 1, 2, 3
