# Supplementary material for: Targeted Suppression of Lipoprotein Receptor LSR in Astrocytes Leads to Olfactory and Memory Deficits in Mice
Source: Int J Mol Sci. 2022 Feb 12;23(4):2049. doi: 10.3390/ijms23042049 (PMC8878779; doi:10.3390/ijms23042049)
Supplement: Supplementary file 1 [file ijms-23-02049-s001.zip › Figure S9.pptx]

## Slide 1
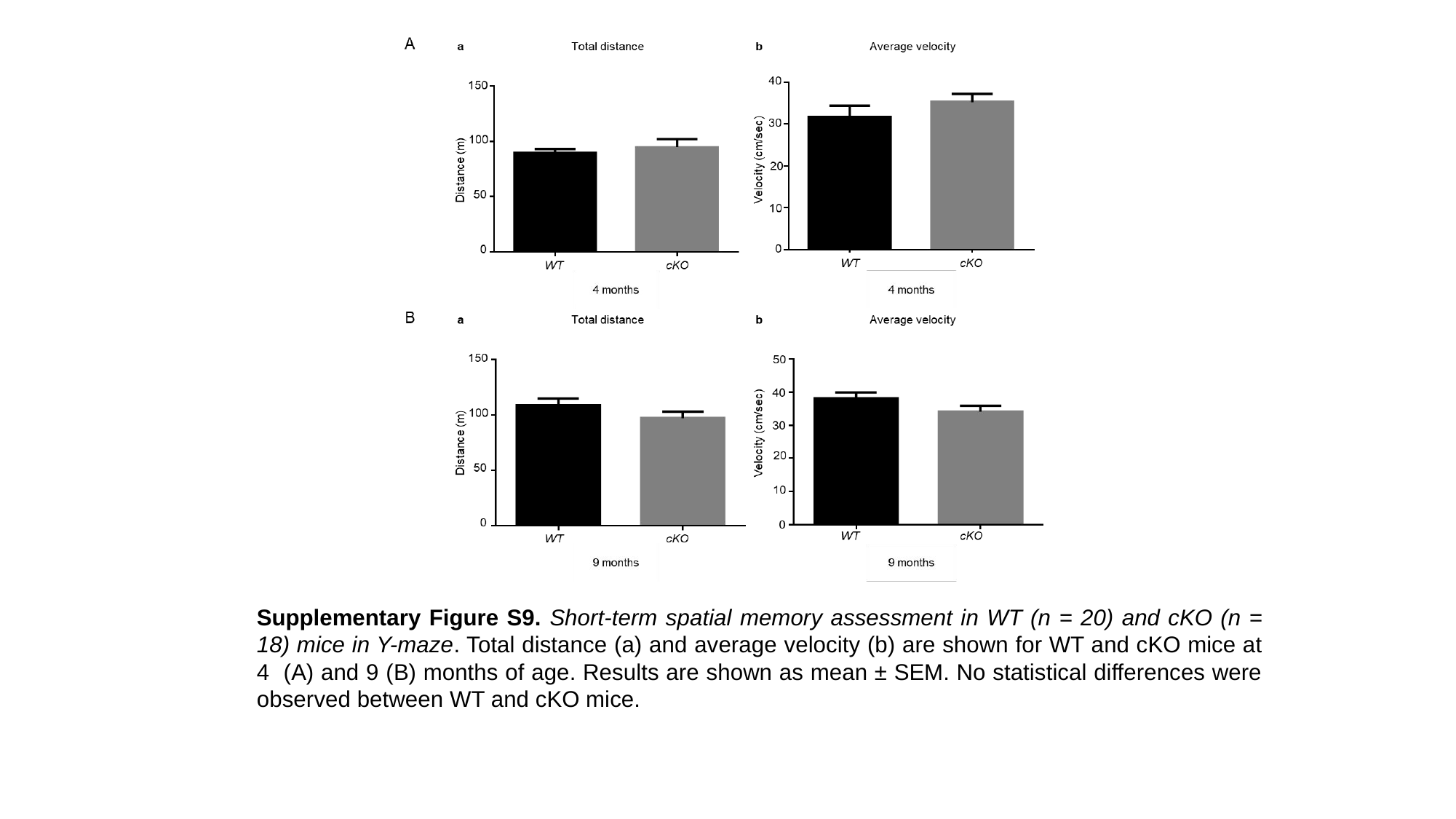

Supplementary Figure S9. Short-term spatial memory assessment in WT (n = 20) and cKO (n = 18) mice in Y-maze. Total distance (a) and average velocity (b) are shown for WT and cKO mice at 4 (A) and 9 (B) months of age. Results are shown as mean ± SEM. No statistical differences were observed between WT and cKO mice.
