# Supplementary material for: Targeted Suppression of Lipoprotein Receptor LSR in Astrocytes Leads to Olfactory and Memory Deficits in Mice
Source: Int J Mol Sci. 2022 Feb 12;23(4):2049. doi: 10.3390/ijms23042049 (PMC8878779; doi:10.3390/ijms23042049)
Supplement: Supplementary file 1 [file ijms-23-02049-s001.zip › Figure S8.pptx]

## Slide 1
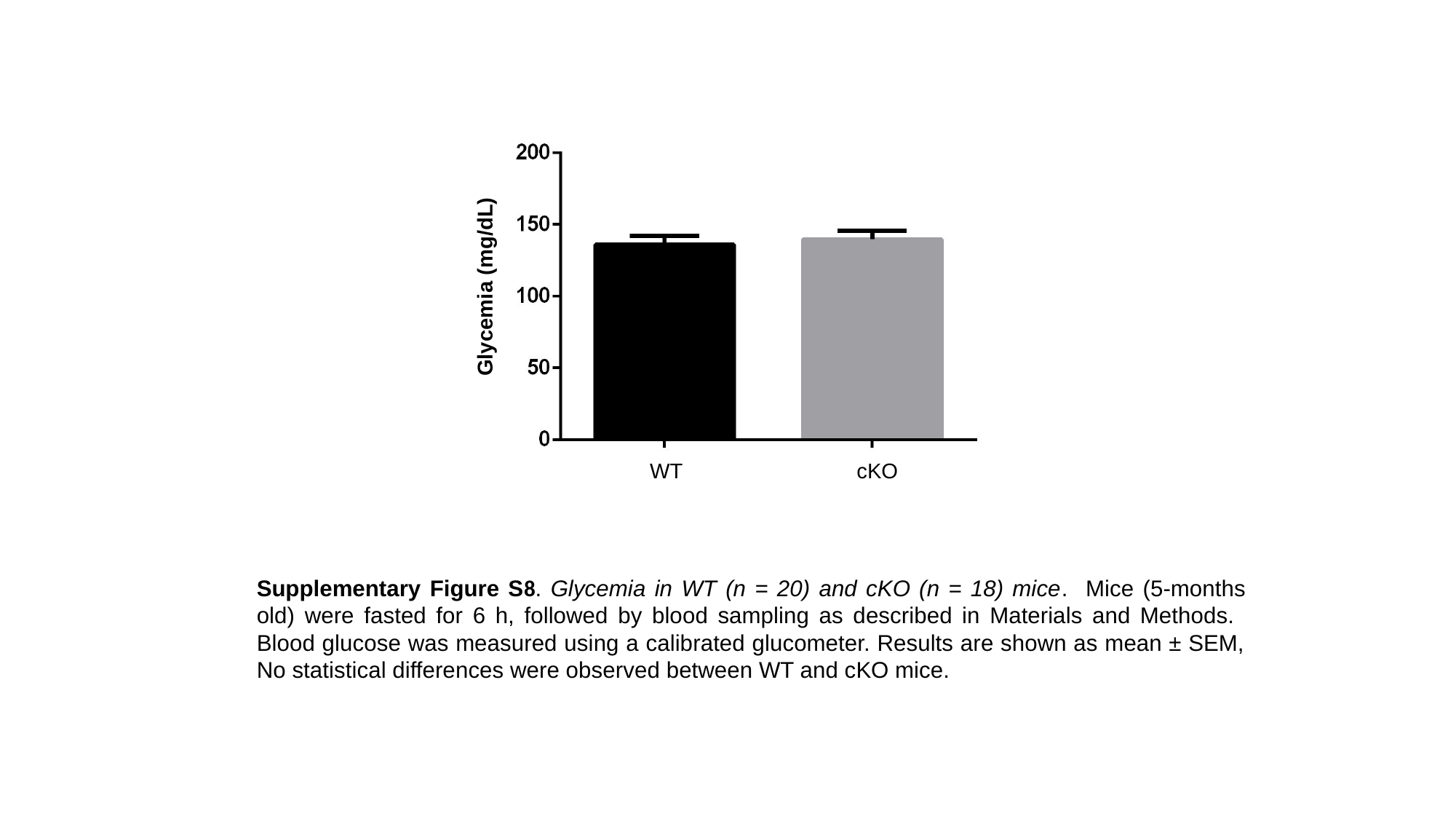

Glycemia (mg/dL)
WT
cKO
Supplementary Figure S8. Glycemia in WT (n = 20) and cKO (n = 18) mice. Mice (5-months old) were fasted for 6 h, followed by blood sampling as described in Materials and Methods. Blood glucose was measured using a calibrated glucometer. Results are shown as mean ± SEM, No statistical differences were observed between WT and cKO mice.
