# Supplementary material for: Targeted Suppression of Lipoprotein Receptor LSR in Astrocytes Leads to Olfactory and Memory Deficits in Mice
Source: Int J Mol Sci. 2022 Feb 12;23(4):2049. doi: 10.3390/ijms23042049 (PMC8878779; doi:10.3390/ijms23042049)
Supplement: Supplementary file 1 [file ijms-23-02049-s001.zip › Figure S7.pptx]

## Slide 1
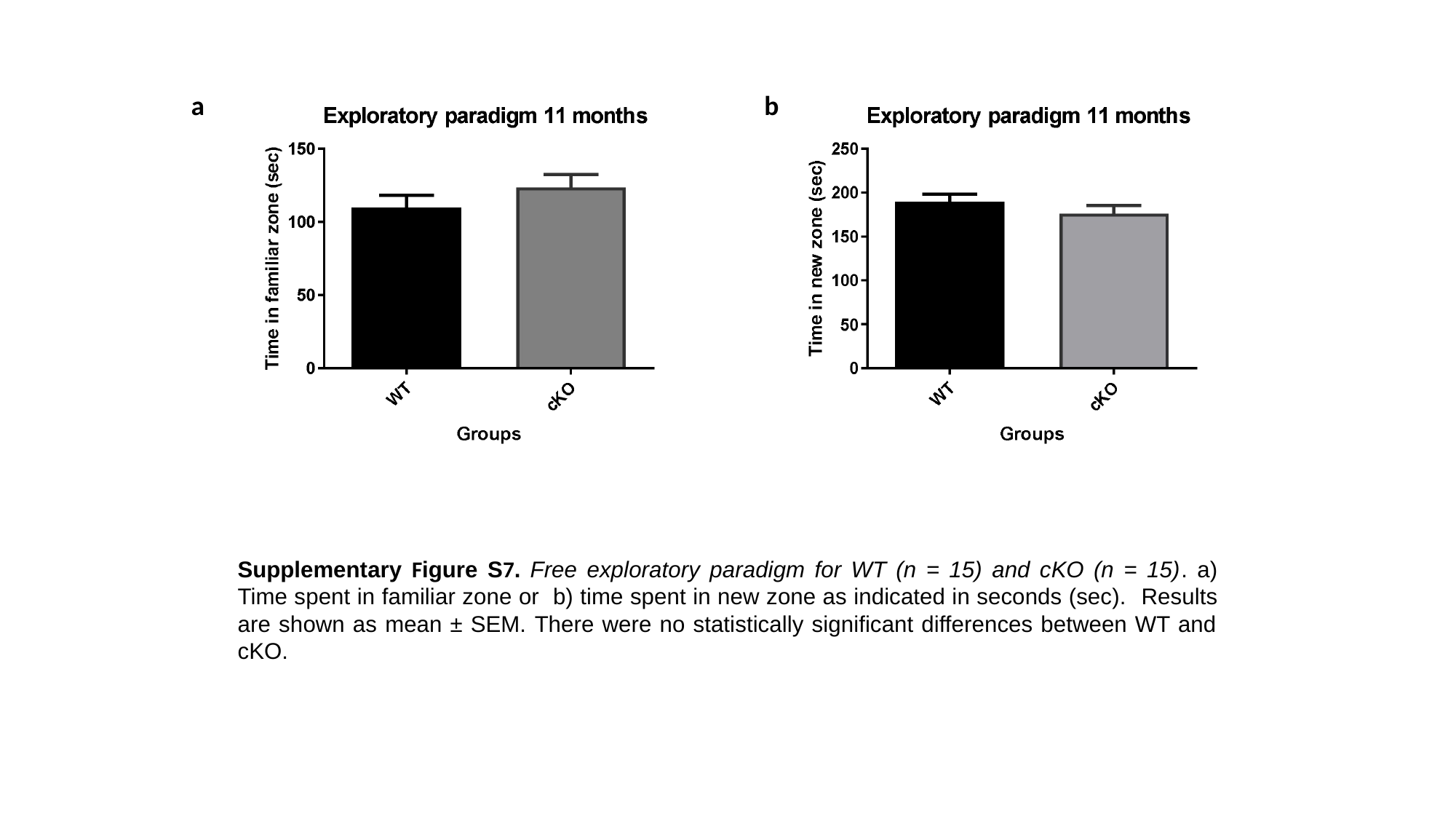

a
b
Supplementary Figure S7. Free exploratory paradigm for WT (n = 15) and cKO (n = 15). a) Time spent in familiar zone or b) time spent in new zone as indicated in seconds (sec). Results are shown as mean ± SEM. There were no statistically significant differences between WT and cKO.
