# Supplementary material for: Targeted Suppression of Lipoprotein Receptor LSR in Astrocytes Leads to Olfactory and Memory Deficits in Mice
Source: Int J Mol Sci. 2022 Feb 12;23(4):2049. doi: 10.3390/ijms23042049 (PMC8878779; doi:10.3390/ijms23042049)
Supplement: Supplementary file 1 [file ijms-23-02049-s001.zip › Figure S6.pptx]

## Slide 1
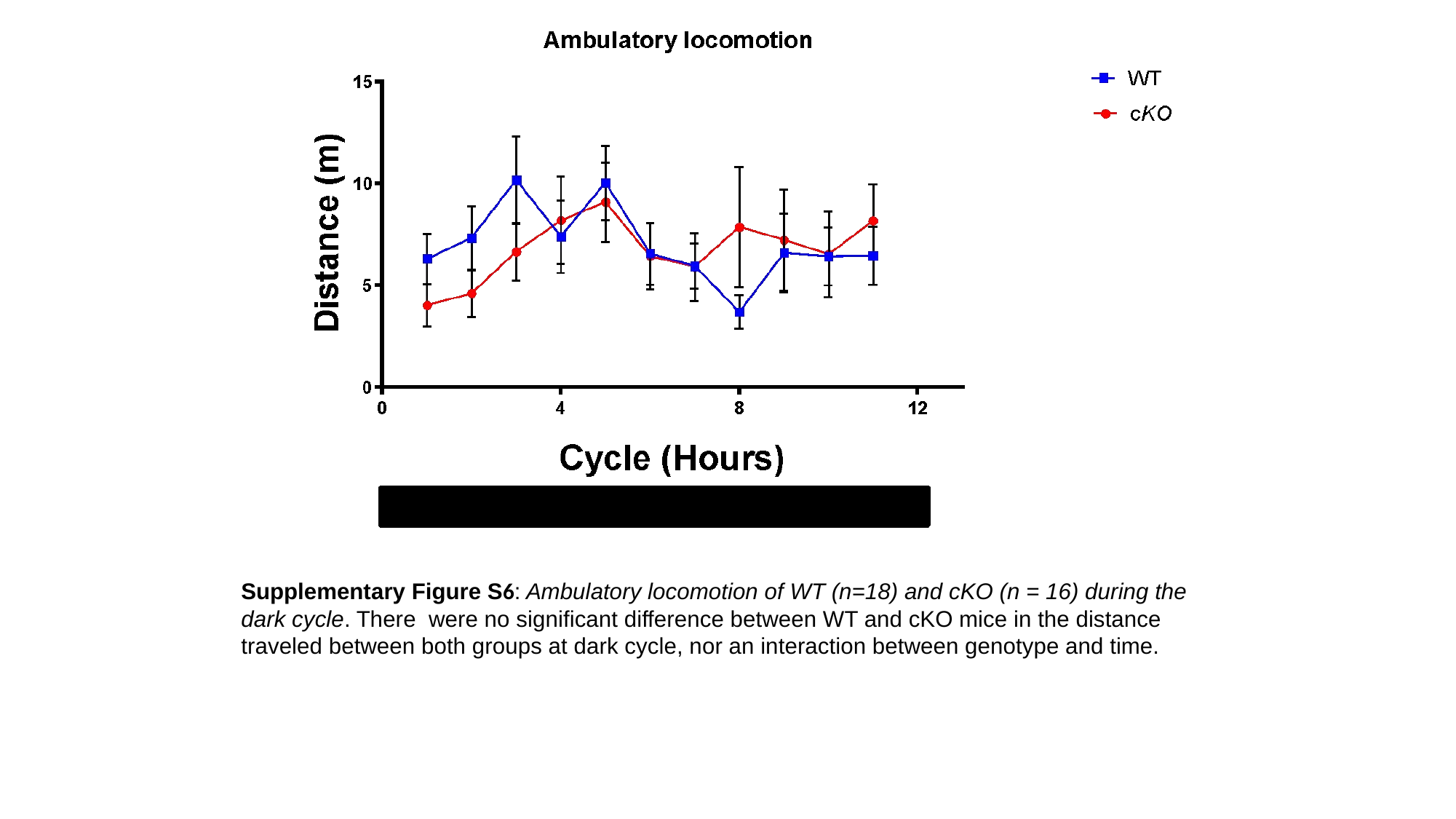

Supplementary Figure S6: Ambulatory locomotion of WT (n=18) and cKO (n = 16) during the dark cycle. There were no significant difference between WT and cKO mice in the distance traveled between both groups at dark cycle, nor an interaction between genotype and time.
