# Supplementary material for: Targeted Suppression of Lipoprotein Receptor LSR in Astrocytes Leads to Olfactory and Memory Deficits in Mice
Source: Int J Mol Sci. 2022 Feb 12;23(4):2049. doi: 10.3390/ijms23042049 (PMC8878779; doi:10.3390/ijms23042049)
Supplement: Supplementary file 1 [file ijms-23-02049-s001.zip › Figure S10.pptx]

## Slide 1
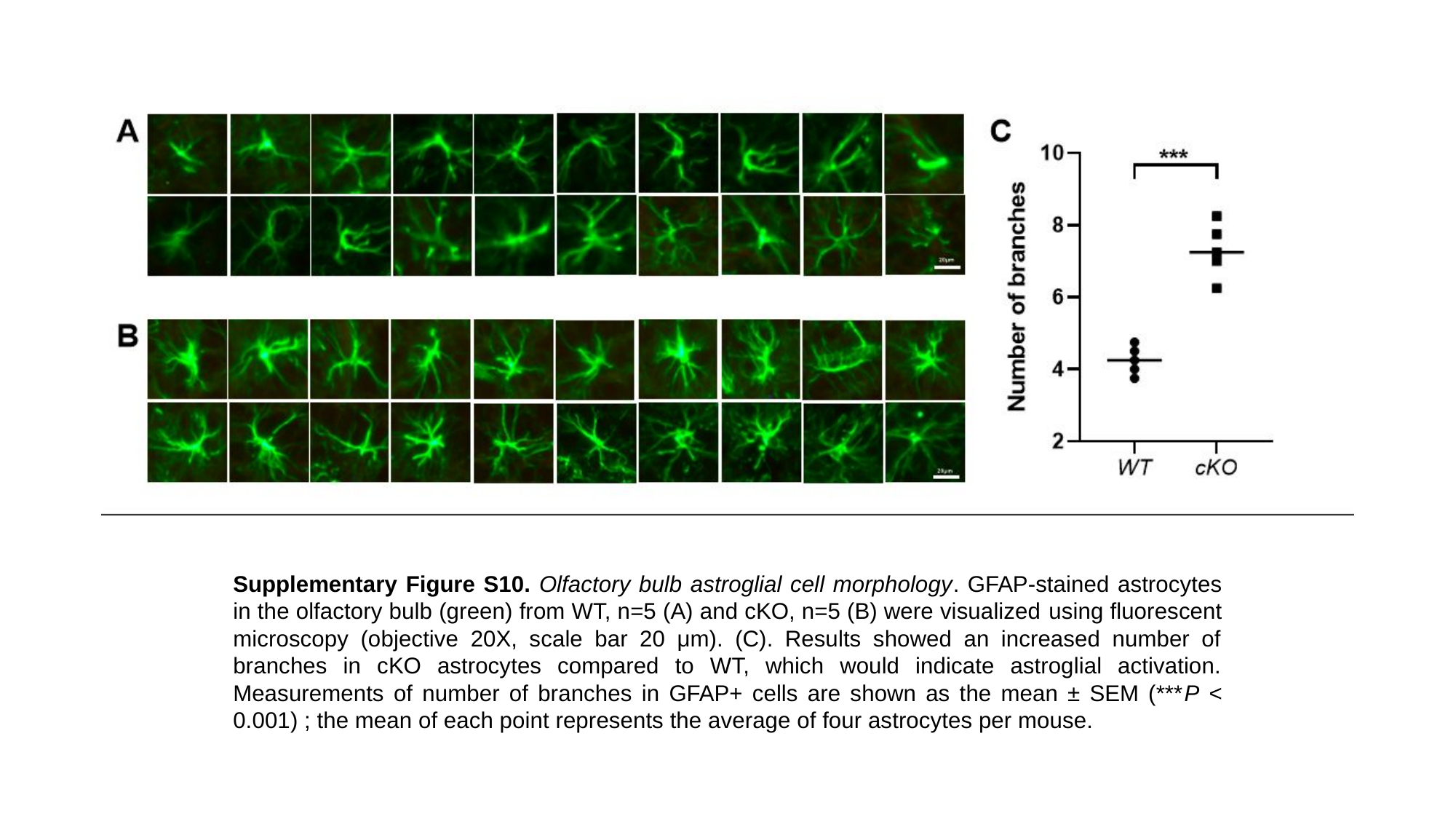

Supplementary Figure S10. Olfactory bulb astroglial cell morphology. GFAP-stained astrocytes in the olfactory bulb (green) from WT, n=5 (A) and cKO, n=5 (B) were visualized using fluorescent microscopy (objective 20X, scale bar 20 μm). (C). Results showed an increased number of branches in cKO astrocytes compared to WT, which would indicate astroglial activation. Measurements of number of branches in GFAP+ cells are shown as the mean ± SEM (***P < 0.001) ; the mean of each point represents the average of four astrocytes per mouse.
