# Supplementary material for: Targeted Suppression of Lipoprotein Receptor LSR in Astrocytes Leads to Olfactory and Memory Deficits in Mice
Source: Int J Mol Sci. 2022 Feb 12;23(4):2049. doi: 10.3390/ijms23042049 (PMC8878779; doi:10.3390/ijms23042049)
Supplement: Supplementary file 1 [file ijms-23-02049-s001.zip › Figure S5.pptx]

## Slide 1
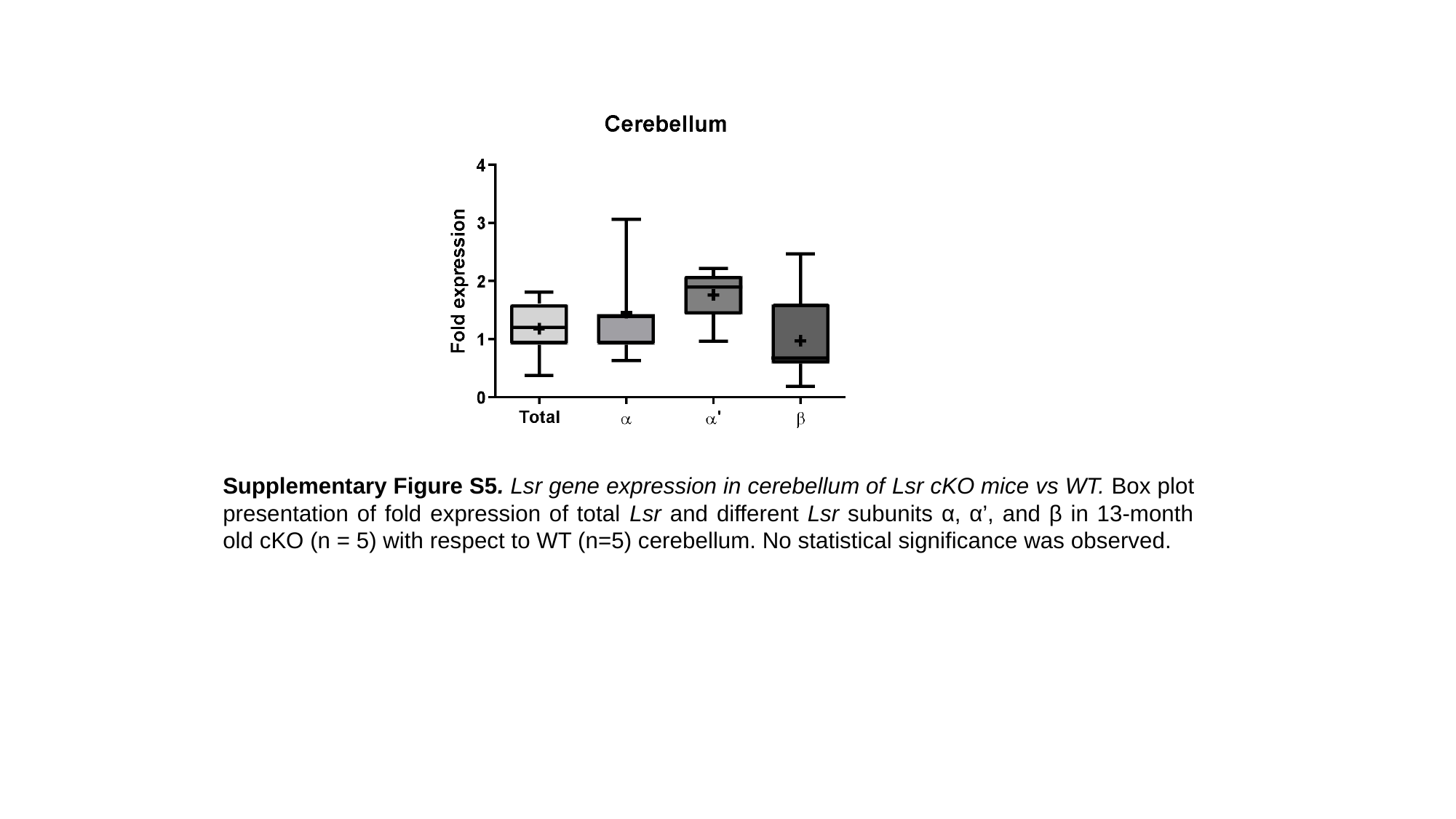

Supplementary Figure S5. Lsr gene expression in cerebellum of Lsr cKO mice vs WT. Box plot presentation of fold expression of total Lsr and different Lsr subunits α, α’, and β in 13-month old cKO (n = 5) with respect to WT (n=5) cerebellum. No statistical significance was observed.
