# Supplementary material for: Targeted Suppression of Lipoprotein Receptor LSR in Astrocytes Leads to Olfactory and Memory Deficits in Mice
Source: Int J Mol Sci. 2022 Feb 12;23(4):2049. doi: 10.3390/ijms23042049 (PMC8878779; doi:10.3390/ijms23042049)
Supplement: Supplementary file 1 [file ijms-23-02049-s001.zip › Figure S4.pptx]

## Slide 1
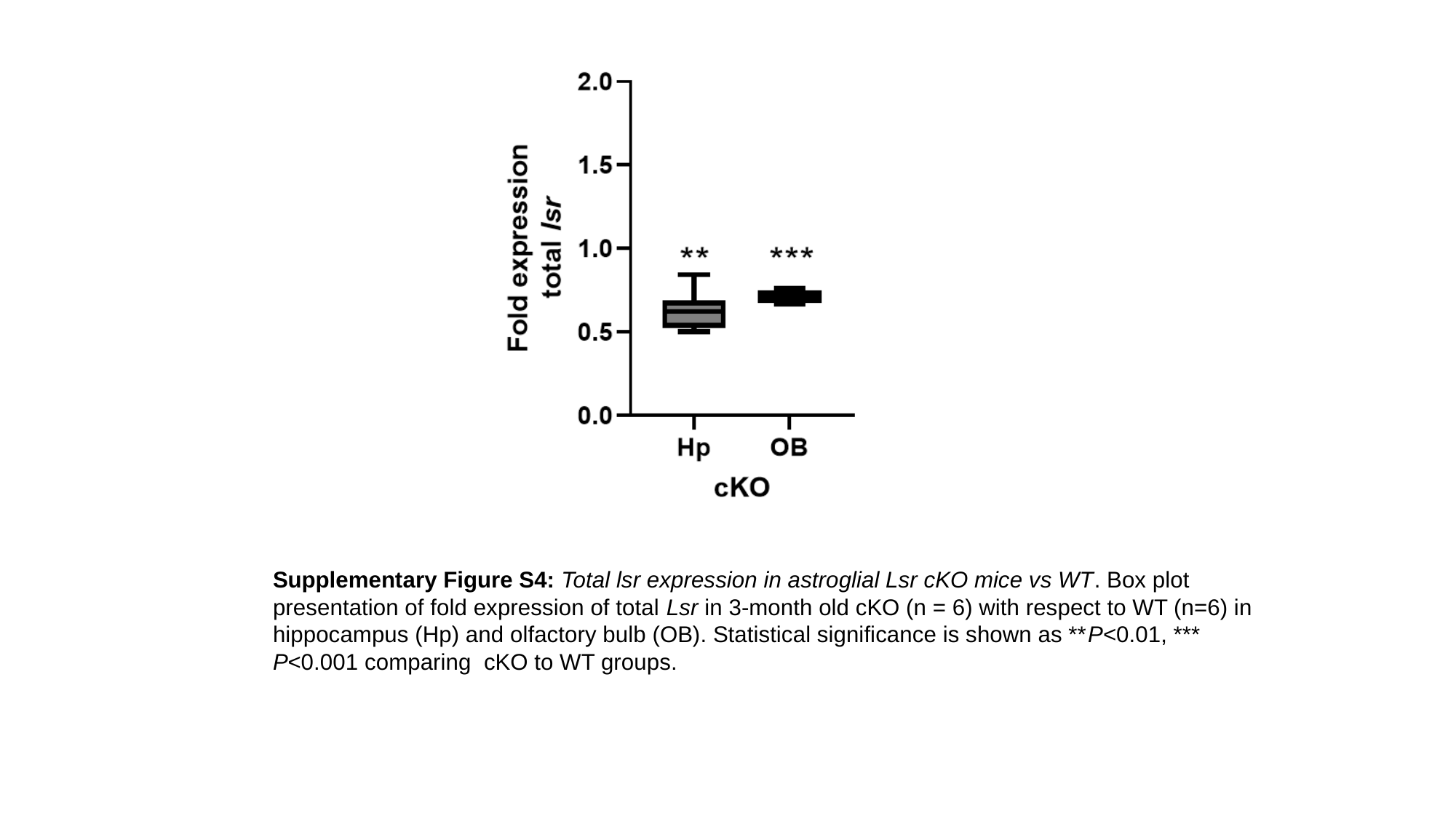

Supplementary Figure S4: Total lsr expression in astroglial Lsr cKO mice vs WT. Box plot presentation of fold expression of total Lsr in 3-month old cKO (n = 6) with respect to WT (n=6) in hippocampus (Hp) and olfactory bulb (OB). Statistical significance is shown as **P<0.01, *** P<0.001 comparing cKO to WT groups.
