# Supplementary material for: Targeted Suppression of Lipoprotein Receptor LSR in Astrocytes Leads to Olfactory and Memory Deficits in Mice
Source: Int J Mol Sci. 2022 Feb 12;23(4):2049. doi: 10.3390/ijms23042049 (PMC8878779; doi:10.3390/ijms23042049)
Supplement: Supplementary file 1 [file ijms-23-02049-s001.zip › Figure S3.pptx]

## Slide 1
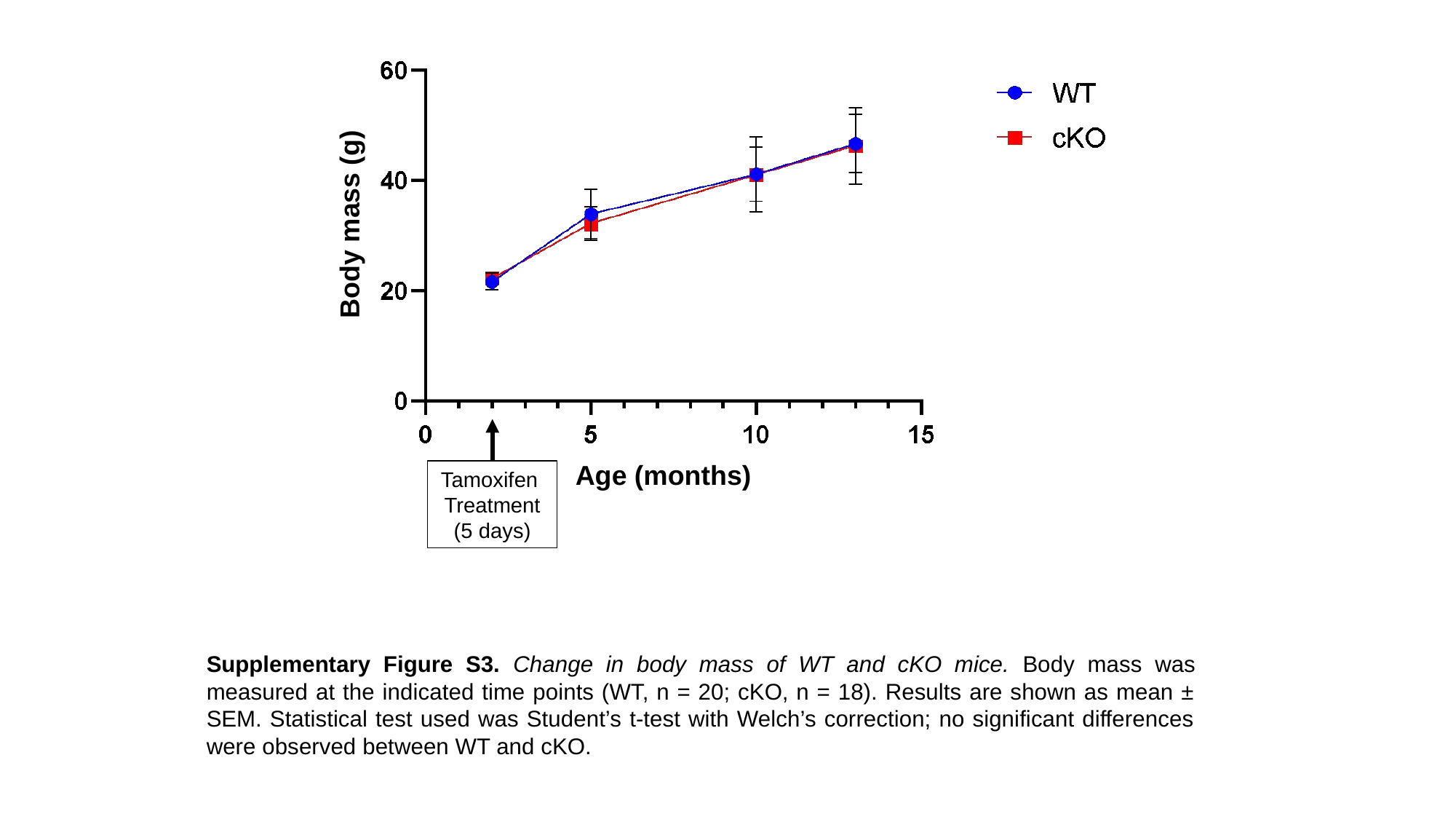

Body mass (g)
Age (months)
Tamoxifen
Treatment (5 days)
Supplementary Figure S3. Change in body mass of WT and cKO mice. Body mass was measured at the indicated time points (WT, n = 20; cKO, n = 18). Results are shown as mean ± SEM. Statistical test used was Student’s t-test with Welch’s correction; no significant differences were observed between WT and cKO.
