# Supplementary material for: Targeted Suppression of Lipoprotein Receptor LSR in Astrocytes Leads to Olfactory and Memory Deficits in Mice
Source: Int J Mol Sci. 2022 Feb 12;23(4):2049. doi: 10.3390/ijms23042049 (PMC8878779; doi:10.3390/ijms23042049)
Supplement: Supplementary file 1 [file ijms-23-02049-s001.zip › Figure S11.pptx]

## Slide 1
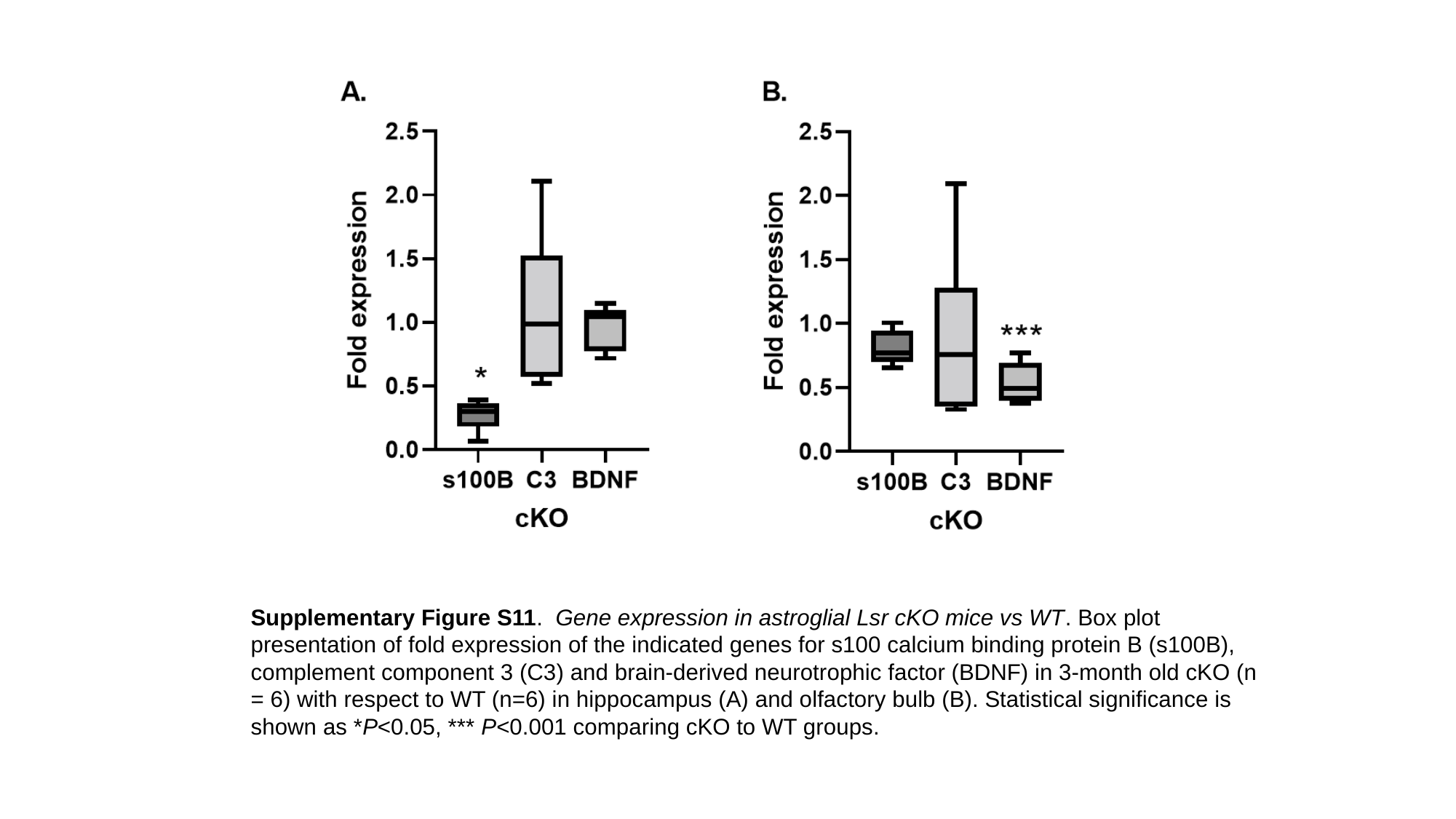

Supplementary Figure S11. Gene expression in astroglial Lsr cKO mice vs WT. Box plot presentation of fold expression of the indicated genes for s100 calcium binding protein B (s100B), complement component 3 (C3) and brain-derived neurotrophic factor (BDNF) in 3-month old cKO (n = 6) with respect to WT (n=6) in hippocampus (A) and olfactory bulb (B). Statistical significance is shown as *P<0.05, *** P<0.001 comparing cKO to WT groups.
