# Supplementary material for: Targeted Suppression of Lipoprotein Receptor LSR in Astrocytes Leads to Olfactory and Memory Deficits in Mice
Source: Int J Mol Sci. 2022 Feb 12;23(4):2049. doi: 10.3390/ijms23042049 (PMC8878779; doi:10.3390/ijms23042049)
Supplement: Supplementary file 1 [file ijms-23-02049-s001.zip › Figure S2.pptx]

## Slide 1
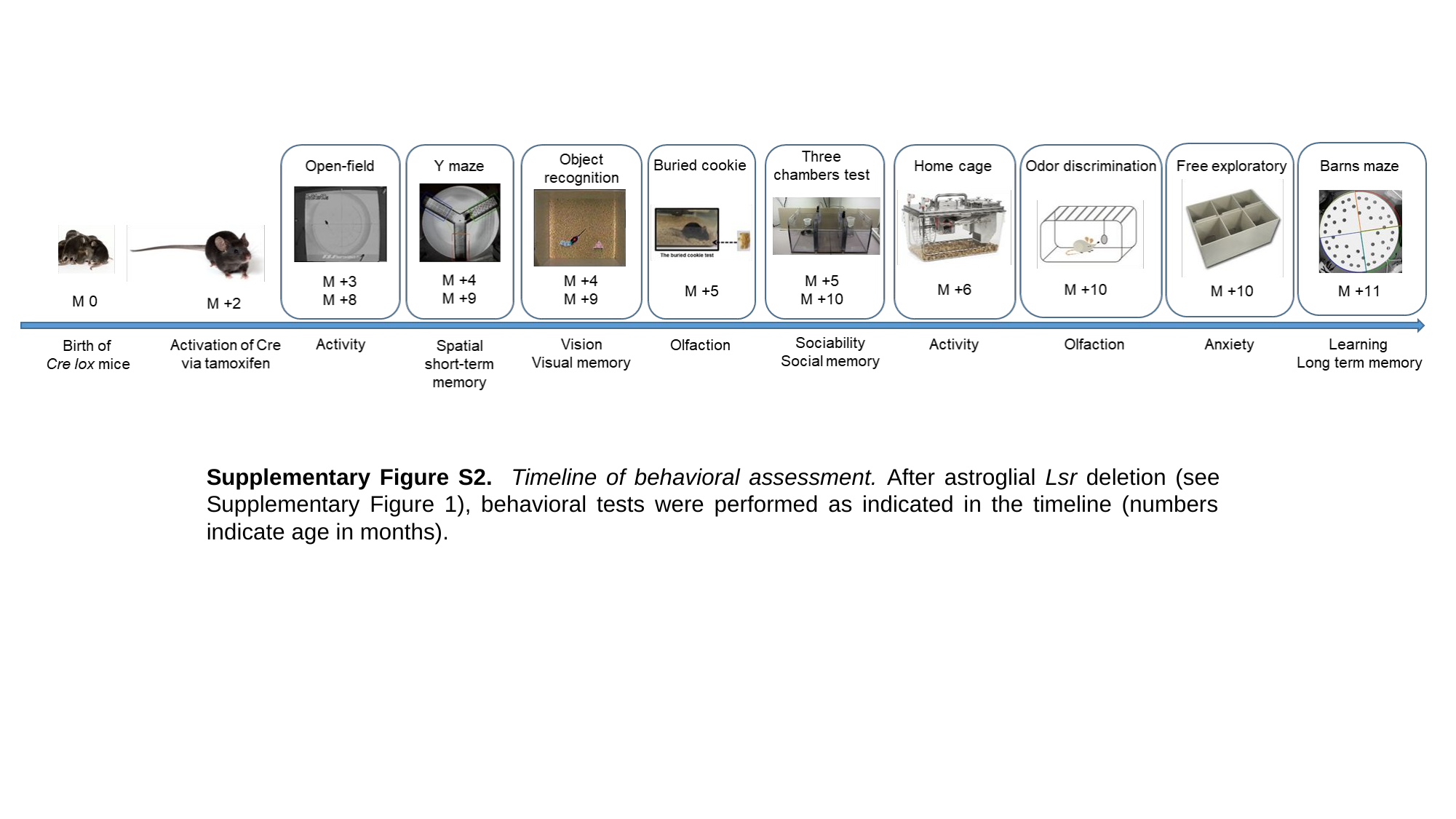

Supplementary Figure S2. Timeline of behavioral assessment. After astroglial Lsr deletion (see Supplementary Figure 1), behavioral tests were performed as indicated in the timeline (numbers indicate age in months).
