# Supplementary material for: Targeted Suppression of Lipoprotein Receptor LSR in Astrocytes Leads to Olfactory and Memory Deficits in Mice
Source: Int J Mol Sci. 2022 Feb 12;23(4):2049. doi: 10.3390/ijms23042049 (PMC8878779; doi:10.3390/ijms23042049)
Supplement: Supplementary file 1 [file ijms-23-02049-s001.zip › Figure S1.pptx]

## Slide 1
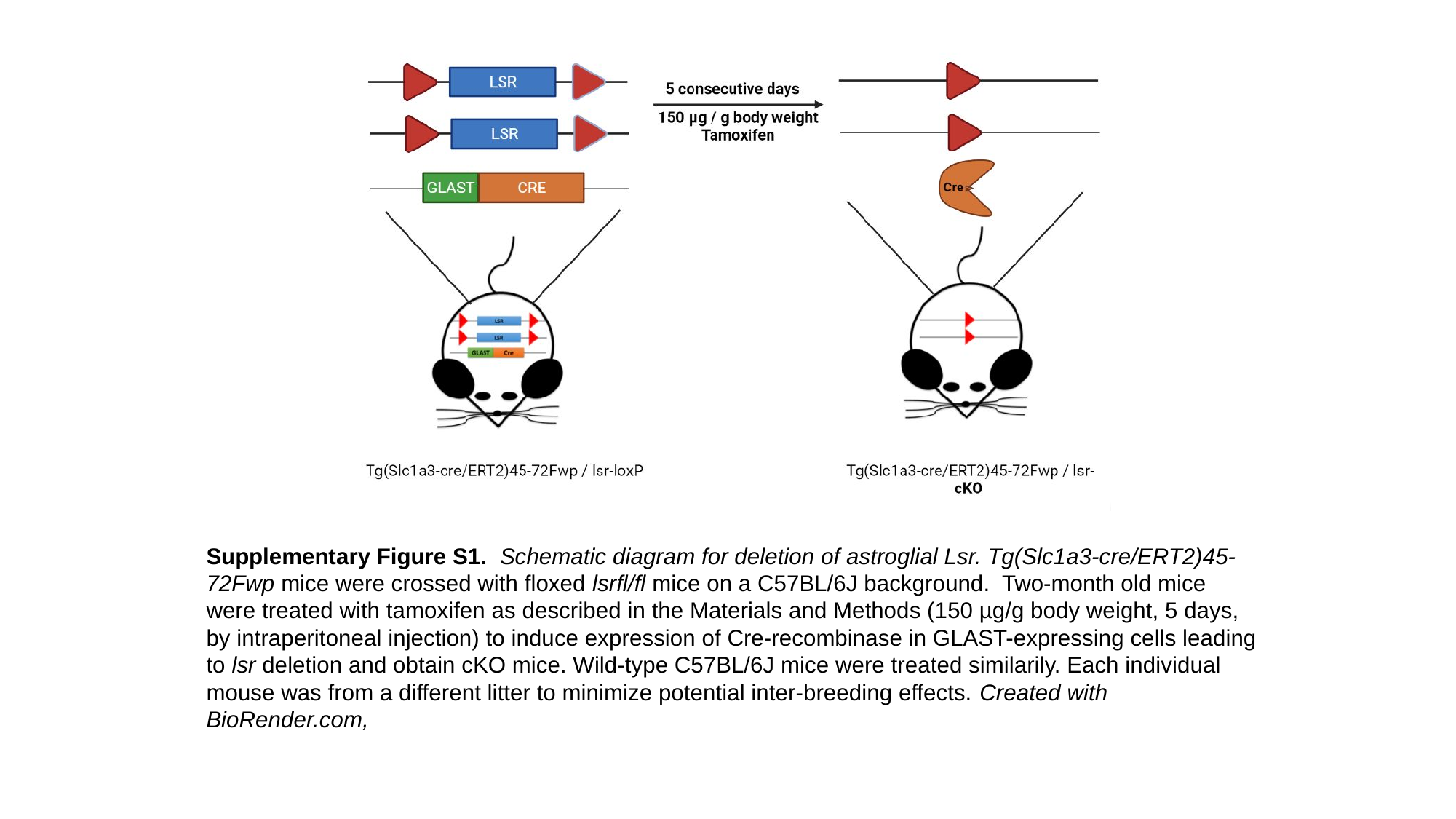

Supplementary Figure S1. Schematic diagram for deletion of astroglial Lsr. Tg(Slc1a3-cre/ERT2)45-72Fwp mice were crossed with floxed lsrfl/fl mice on a C57BL/6J background. Two-month old mice were treated with tamoxifen as described in the Materials and Methods (150 µg/g body weight, 5 days, by intraperitoneal injection) to induce expression of Cre-recombinase in GLAST-expressing cells leading to lsr deletion and obtain cKO mice. Wild-type C57BL/6J mice were treated similarily. Each individual mouse was from a different litter to minimize potential inter-breeding effects. Created with BioRender.com,
