# Supplementary material for: Targeted Suppression of Lipoprotein Receptor LSR in Astrocytes Leads to Olfactory and Memory Deficits in Mice
Source: Int J Mol Sci. 2022 Feb 12;23(4):2049. doi: 10.3390/ijms23042049 (PMC8878779; doi:10.3390/ijms23042049)
Supplement: Supplementary file 1 [file ijms-23-02049-s001.zip › Table S1.pptx]

## Slide 1
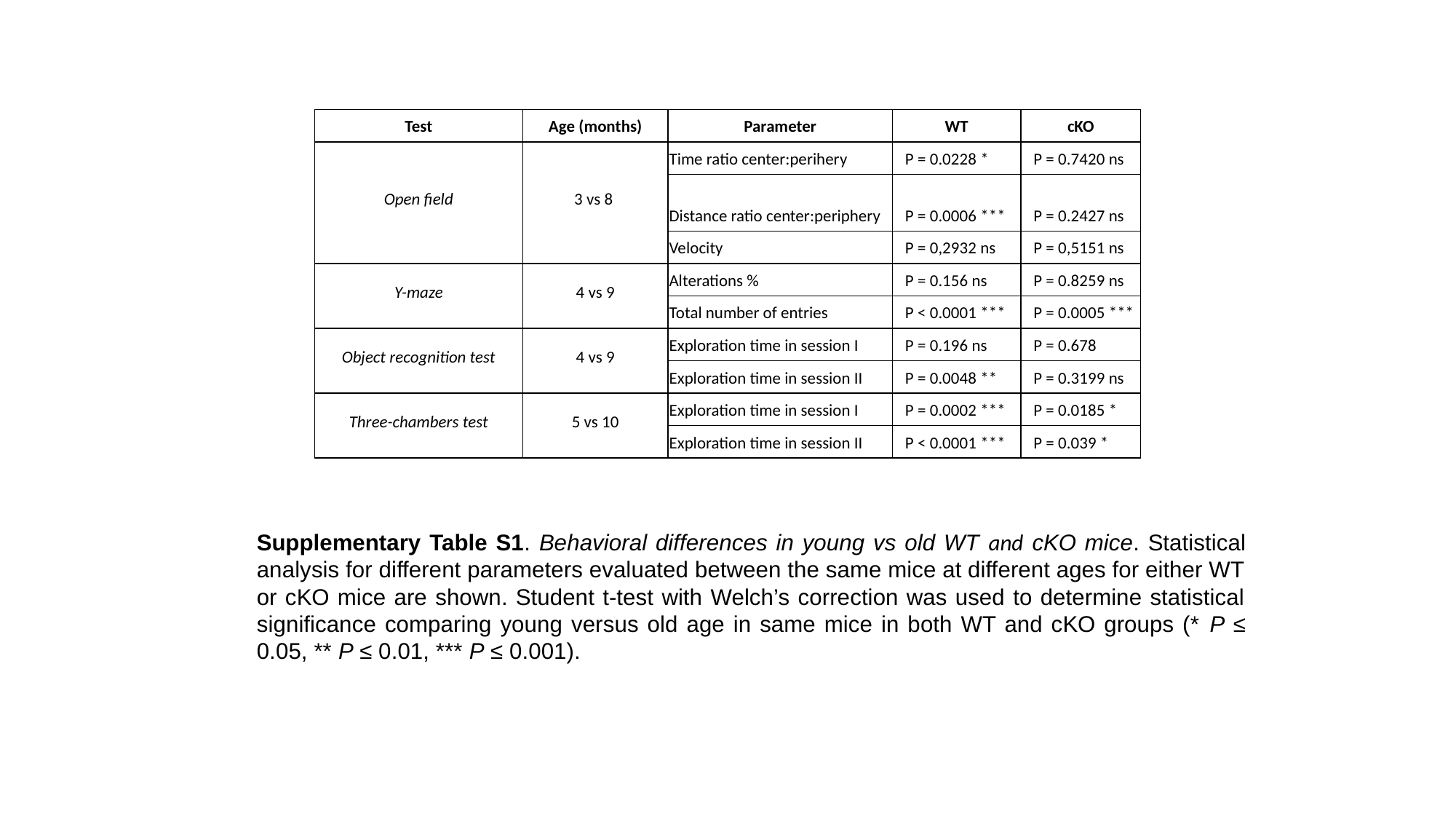

| Test | Age (months) | Parameter | WT | cKO |
| --- | --- | --- | --- | --- |
| Open field | 3 vs 8 | Time ratio center:perihery | P = 0.0228 \* | P = 0.7420 ns |
| | | Distance ratio center:periphery | P = 0.0006 \*\*\* | P = 0.2427 ns |
| | | Velocity | P = 0,2932 ns | P = 0,5151 ns |
| Y-maze | 4 vs 9 | Alterations % | P = 0.156 ns | P = 0.8259 ns |
| | | Total number of entries | P < 0.0001 \*\*\* | P = 0.0005 \*\*\* |
| Object recognition test | 4 vs 9 | Exploration time in session I | P = 0.196 ns | P = 0.678 |
| | | Exploration time in session II | P = 0.0048 \*\* | P = 0.3199 ns |
| Three-chambers test | 5 vs 10 | Exploration time in session I | P = 0.0002 \*\*\* | P = 0.0185 \* |
| | | Exploration time in session II | P < 0.0001 \*\*\* | P = 0.039 \* |
Supplementary Table S1. Behavioral differences in young vs old WT and cKO mice. Statistical analysis for different parameters evaluated between the same mice at different ages for either WT or cKO mice are shown. Student t-test with Welch’s correction was used to determine statistical significance comparing young versus old age in same mice in both WT and cKO groups (* P ≤ 0.05, ** P ≤ 0.01, *** P ≤ 0.001).
